# Evolution of cooperation in multiplex networks through asymmetry between interaction and replacement

**DOI:** 10.1038/s41598-023-37074-4

**Published:** 2023-06-17

**Authors:** Masaaki Inaba, Eizo Akiyama

**Affiliations:** Graduate School of Systems and Information Engineering, University of Tsukuba, Tsukuba, Japan

**Keywords:** Complex networks, Evolutionary theory

## Abstract

Cooperation is the foundation of society and has been the subject of numerous studies over the past three decades. However, the mechanisms underlying the spread of cooperation within a group are not yet fully comprehended. We analyze cooperation in multiplex networks, a model that has recently gained attention for successfully capturing certain aspects of human social connections. Previous studies on the evolution of cooperation in multiplex networks have shown that cooperative behavior is promoted when the two key processes in evolution, interaction and strategy replacement, are performed with the same partner as much as possible, that is, symmetrically, in a variety of network structures. We focus on a particular type of symmetry, namely, symmetry in the scope of communication, to investigate whether cooperation is promoted or hindered when interactions and strategy replacements have different scopes. Through multiagent simulations, we found some cases where asymmetry can promote cooperation, contrasting with previous studies. These results hint toward the potential effectiveness of not only symmetrical but also asymmetrical approaches in fostering cooperation within particular groups under certain social conditions.

## Introduction

Many living organisms, including humans, have survived in harsh environments by forming groups and by cooperating with each other within those groups to protect themselves from external enemies. However, it is common for individuals to behave selfishly, which can lead to conflicts within a group and can make cooperation difficult. This is because when individuals prioritize their own interests, acting as so-called free riders, who receive cooperation from others only and do not cooperate with others themselves, have an advantage, at least in the short term. The type of cooperative behavior we refer to here is altruism. There is still much that we do not know about the conditions necessary for establishing and maintaining cooperative relationships, which is the subject of research in the area of the evolution of cooperation. Many mechanisms^1–3^ for the evolution of cooperation have been studied within the framework of evolutionary game theory^4^. Among these mechanisms, our study is focused on the mechanism of network reciprocity^5–10^, which considers the differences in the structure of connections among individuals, and among network reciprocity mechanisms, we specifically examine the multiplex network.

Network reciprocity^5–10^ is a mechanism in which the connections between individuals are represented as a network, and the differences in network structure influence the spread of cooperation. One of the important findings regarding the evolution of cooperation in networks, which is closely related to our study, is that cooperation is more likely to evolve in scale-free networks than in regular or random networks^5,6^. A scale-free network is a network structure with a power-law degree distribution, a shorter average distance, and a higher average clustering coefficient, and it is similar to the network structures commonly found in human societies^11–14^. The reason cooperation is more likely to evolve in scale-free networks is that cooperative individuals around hubs, which are individuals with much higher degrees of connection than others, can enhance each other’s fitness, which enables the cooperative strategy to spread throughout the population. Cooperators benefit from forming groups around hubs, whereas defectors have no incentive to form groups since they exploit each other. Such a difference between cooperators and defectors, along with the presence of hubs in scale-free networks, promotes the evolution of cooperation. Regular networks lack hubs, and random networks have small or few hubs. Thus, scale-free networks are more likely to facilitate the evolution of cooperation than regular or random networks.

It should be noted that the findings mentioned above were obtained from a model that begins with a certain portion of the population as cooperators. On the other hand, there exist studies that analyze the evolution of cooperative behavior by using fixation probabilities, i.e., the likelihood of one individual’s strategy dominating the entire population, starting from a state in which only a single individual in the population is a cooperator^15–20^. These studies, using fixation probabilities, facilitate strict mathematical analysis under the weak selection condition, leading to numerous well-established research conclusions. Some of these findings suggest that network structural heterogeneity may inhibit cooperation. However, in models that start with a single individual, the mechanism we have described, where cooperators form clusters on a scale-free network to promote cooperation, is not observed. This is because if there is only one cooperator at the start, there are no clusters to foster cooperation, and the cooperator likely perishes before forming a cluster. Therefore, investigating the evolution of cooperation in models starting from many individuals such as ours, which cannot be reproduced in a model starting from a single individual, is beneficial as a complement to each other even in the recent situation in which much meaningful research is being done on mathematical models with fixed probabilities based on the assumption of weak selection.

While many network reciprocity studies assume that interactions and strategy replacements are constrained to a single network, we address the evolution of cooperation in two-layer multiplex networks^3,15,16,18,21–23^, where interactions and replacements are constrained to two different networks. A multiplex network is a network consisting of multiple layers, where nodes are common to all layers while edges are connected in different ways in each layer. A similar concept is the multilayer network^3,24^, in which nodes are not necessarily common to all layers. Multiplex networks are suitable for representing and for analyzing how people belong to different relationships and how they perform different activities in each relationship^22^. Interactions are game-theoretic games that cause an increase or decrease in each individual’s payoff, and strategy replacements are behaviors that cause a change in each individual’s behavioral traits, such as reproduction, imitation, or learning. The evolution of cooperation is often modeled by the repetition of these two activities. Although general multiplex networks can have three or more layers, studies on the evolution of cooperation in multiplex networks typically consider a two-layer multiplex network, with one network for each of these two activities.

The evolution of cooperation in two-layer multiplex networks, where the interaction network constrains the choice of the partner in the interaction and the replacement network constrains the choice of the parent or imitator for the strategy replacement, has been actively studied^15,16,18,22^. For example, Ohtsuki^16^ conducted an analytical study on two-layer regular networks, and Wang^22^ conducted a simulation study on two-layer scale-free networks. Both studies indicated that the higher the symmetry of both networks, the more cooperative behavior is promoted. Similarly, Su^18^ conducted analytical and simulation studies on two-layer dynamic weighted networks and essentially concluded, as in the previous two studies, that symmetry in both networks promotes cooperation. Additionally, that study addressed two types of symmetry in public goods games on dynamic weighted networks: symmetry of network structure and symmetry of partner selection for interactions and strategy replacements. The authors showed that the asymmetry of network structure and the symmetry of partner selection promote cooperation. Therefore, it is also reasonable to regard this aspect as strengthening the robustness of the conclusions of the previous two studies, indicating that cooperation is more likely to evolve if the interactions and replacements are conducted with the same partner as much as possible. Several prior studies^15,16,18,22^ have essentially led to the same conclusion that symmetry promotes cooperation, even though each was studied under very different assumptions. Thus, showing that this conclusion holds in general, or finding exceptions to it and showing that it does not, is an important concern in the study of the evolution of cooperation in multiplex networks.

Our study is focused on one particular symmetry, which is the symmetry of scope on communication. We examine whether cooperation is facilitated or impeded when interactions and strategy replacements have different scopes. If the conclusion of previous studies^15,16,18,22^ that interactions and replacements with the same partner promote cooperation applies to this situation as well, then it follows that both interaction and replacement with the same scope promote more cooperation. Our interest is motivated by the argument that, in an ecological context^25^, cooperative behavior evolves more when individuals interact locally but learn globally. To represent this locality and globality, we implement a method of extending networks. The focus of our study is on scale-free networks, which are relatively close to the network structures commonly found in human society. However, we also analyze regular and random networks for comparative purposes. We examine whether the conclusions of previous studies, which have been verified for various network structures, hold for the network structure of our study, which exhibits asymmetry in the scope of communication. After examining whether the conclusions of the previous studies apply to our network structure, we find that scope asymmetry can promote cooperation in some cases. Specifically, we show that having a local scope of interaction and a global scope of strategic replacements promotes cooperation. Although scope symmetry is a concept encompassed by network symmetry, this finding has not been mentioned in prior research, and it may provide an opportunity to deepen the basic theory of the evolution of cooperation in multiplex networks.

## Model

We model the evolution of cooperation in multiplex networks consisting of two layers, namely, an interaction network and a replacement network, by using a multiagent simulation. Agents are represented as nodes in the networks, and the relationships between agents are represented as edges. Initially, agents are assigned two strategies, cooperation and defection, with a 50–50 probability. Then, the agents connected in the interaction network interact with each other, and the agents connected in the strategy replacement network update their strategies, repeating this process for many generations. If the frequency of cooperators increases as a result, we consider cooperation to have evolved. Since our purpose is to investigate how differences in network structure affect the evolution of cooperation, the network construction method is the unique part of this model, while the interaction and replacement rules follow the standard methods used in many previous studies^7,15,16,18,22,26–29^.

### Network structure

As introduced earlier, we employ a multiplex network to represent the local-global scope of communication. This multiplex network is constructed by stacking two *h*-hop extension networks. The *h*-hop extension of the base network, comprising *N* nodes, is constructed as follows: First, the base network is replicated as the initial state of the extension network. Nodes in both networks are assigned identification numbers ranging from 1 to N. Next, for nodes $a_i$ and $a_j$ (where *i*, $j \in \{1, 2, \ldots , N\}$ and $i < j$), if the shortest distance $d_{i,j}$ between these nodes in the base network is less than or equal to *h*, a new edge is established between $a_i$ and $a_j$ in the extension network. This process is repeated for all possible combinations of *i* and *j*, resulting in an *h*-hop extension network.

**Figure 1:**
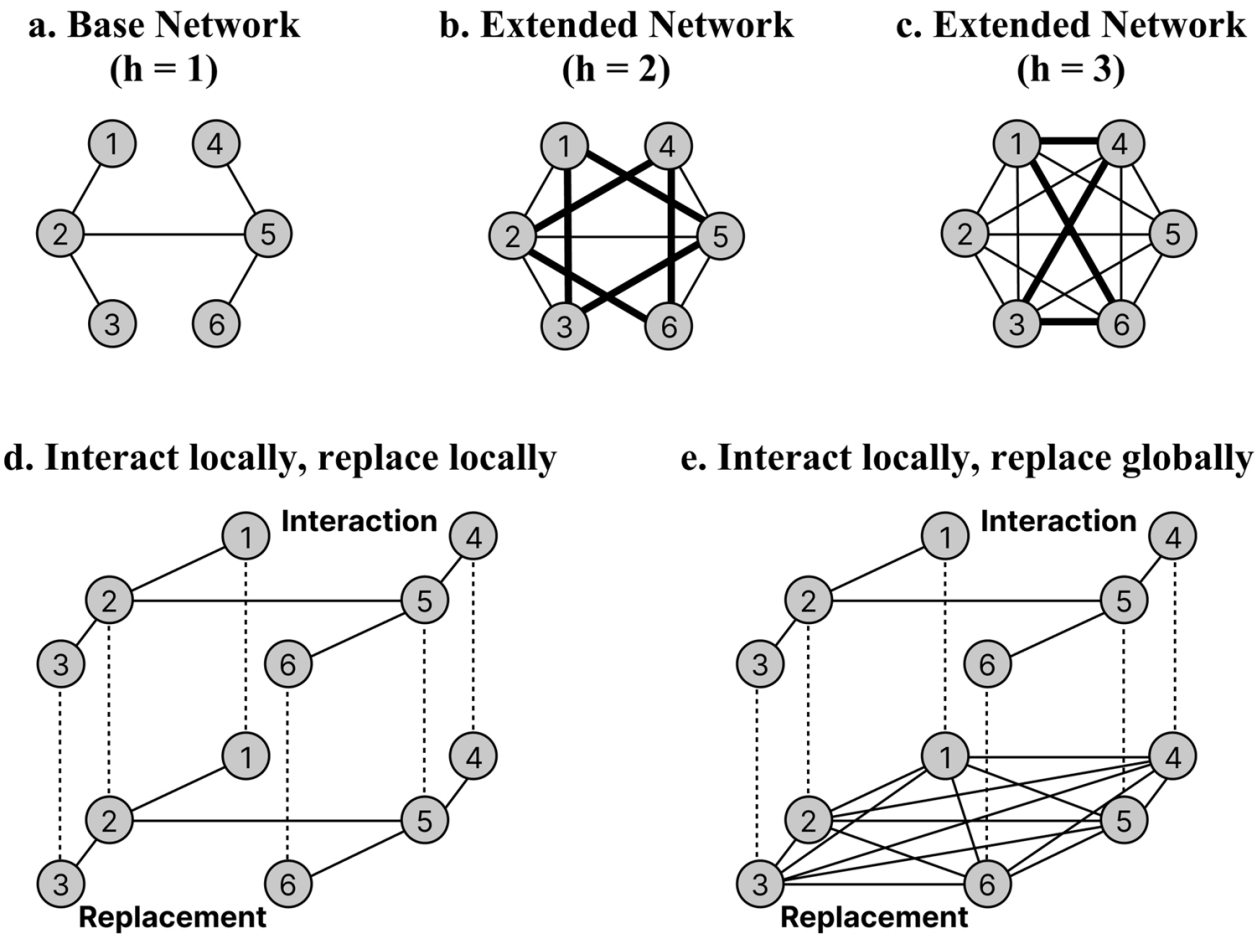
Network expansion method. Starting from the base network (**a**), the network is extended as (**b**) and (**c**). The expansion networks are then combined to form a multiplex network as shown in (**d**) and (**e**).

Figure 1 provides a simple illustrative example. In the base network (Fig. 1a), if there are no edges between nodes that are connected by edges within *h* hops, a new edge is created. For example, Fig. 1b shows a 2-hop extension, and Fig. 1c shows a 3-hop extension. The networks created in this way are combined into a two-layer network, with the upper network representing the interaction network and the lower network representing the strategy replacement network. The interaction network constrains the selection of interaction partners, while the replacement network constrains the selection of replacement partners. Figure 1d shows an example of a symmetric network in which both interaction and replacement are performed within a local scope. Figure 1e shows an example of an asymmetric network in which interactions are local and replacements are global. Although asymmetric multiplex networks can be constructed in many other ways, we focus on this type of asymmetry, specifically the local-global scope of communication. We note that the network structure becomes a complete network at the 3-hop extension, so the network structure does not change even if the extension hops are increased further. We consider only undirected, unweighted, static, and connected networks. Although the simple and small network is shown as the base network in Fig. 1 to convey an idea, the actual simulations use complex networks with more nodes. Additionally, we construct scale-free networks by using the BA model^11,30^ and random networks by using the ER model^11,31^. The Supplemental Information contains the details on the features of the networks constructed through this extension.

### Interaction rule

Interaction is a game-theoretic concept in which an agent’s payoff depends on their own strategy and the strategies of their opponents. We analyze both pairwise and N-player games. Pairwise games are represented by the prisoner’s dilemma (PD), while N-player games are represented by public goods games (PGGs)^15,16,18,22^. Importantly, in both types of games, maintaining a defection strategy results in a higher payoff than that of maintaining a cooperative strategy in a single game.

In the pairwise game, each agent takes turns being the focal agent and plays PD games with all of its neighbors in the interaction network. The payoffs for each game are determined by Table 1^29^ and accumulated over all games in a generation.

**Table 1:** Payoff table.

	Cooperator	Defector
Cooperator	$b-c$	$-c$
Defector	*b*	0

(b > c > 0).

In the N-player game, each agent takes turns being the focal agent and forms a group with all of its neighbors in the interaction network to play the PGG. The PGG proceeds as follows: First, cooperators in the group contribute *c* resources, while defectors contribute nothing. Then, the group’s resources are multiplied by *b*/*c*, and the resulting resources are divided equally among all group members ($b>c>0$). Specifically, if the group consists of *n* agents and $n_C$ of them are cooperators, the cooperator’s payoff is $\pi _C=\frac{b \times n_C}{n} - c$, and the defector’s payoff is $\pi _D=\frac{b \times n_C}{n}$. The PGG is an extension of the PD game to N players^8^.

### Replacement rule

Strategy replacement is an action that causes a change in each individual’s behavioral trait, which corresponds to death or reproduction in the biological context and imitation or learning in the social and cultural context. Replacement is based on the fitness of individuals, with higher fitness making it more likely for the behavioral trait to spread in the population. The fitness is determined by the cumulative payoff, which is a result of interactions. In many studies^26–29^ on the evolution of cooperation, they consider payoff as one of several factors that determine fitness, accounting for a variety of external factors. We adopt the same approach, assuming that the fitness (*f*) can be expressed as $f=1-\delta +\pi \delta$^26–28^, where $\pi$ is the cumulative payoff and $\delta$ is a constant value ($0<\delta <1$) representing the strength of the effect of the cumulative payoff on the fitness. In this study, we consider three types of replacement rules: birth–death (BD), death–birth (DB), and imitation (IM), all of which have been extensively studied^7,16,27^.

In the birth–death (BD) rule, strategy replacement proceeds as follows: (1) One agent is selected as the parent from among all agents based on their fitness. Agents with a high fitness are more likely to be selected, while those with a low fitness are less likely to be chosen. (2) The parent gives birth to a child agent, which inherits its parent’s strategy. (3) One of the agents connected to the parent in the replacement network is randomly selected and removed (i.e., dies). (4) The child replaces the removed agent.

In the death–birth (DB) rule, strategy replacement proceeds as follows: (1) One agent is randomly selected from among all agents and removed. (2) One of the agents connected to the removed agent in the replacement network is selected and becomes the parent agent according to their fitness. Agents with a high fitness are more likely to be selected, and agents with a low fitness are less likely to be selected. (3) The parent agent gives birth to a child agent, and the child inherits its parent’s strategy. (4) The child agent replaces the removed agent.

In the imitation (IM) rule, strategy replacement proceeds as follows: (1) One agent is randomly selected among all agents (Agent *A*). (2) One of the agents connected to *A* in the replacement network is randomly selected (Agent *B*). (3) *A* imitates *B*’s strategy according to the difference in payoffs between them. The pairwise-Fermi function^32,33^ $P_{A \rightarrow B}=\frac{1}{1 + exp(\frac{\pi _A - \pi _B}{\delta })}$ is used to output the imitation probability ($P_{A \rightarrow B}$) from the payoffs ($\pi$), with the selection strength ($\delta$).

To summarize the entire simulation flow, first, the parameters (Table 2) are determined, including the network structure, interaction rules, and strategy replacement rules. Then, we run the simulation for 10,000 generations over 100 trials. During each generation, all agents sequentially execute interactions as a focal agent. The fitnesses are calculated from the payoffs accumulated through the interactions, and replacements are performed. Payoffs and fitnesses are initialized to 0 for each generation. The values that change during the simulation are the strategy, payoff, and fitness of each agent. After the simulation, we calculate the population frequency of cooperators in the last 1000 generations of each trial and average them to obtain the cooperation rate.

**Table 2:** Parameters determined at the beginning of the simulation.

Category	Parameter	Options
Network structure	Base network	Regular ($k=4$), Random ($\bar{k}=4$), Scale-free ($\bar{k}=4$)
	$h_G$: Expansion hop count of interaction network	1, ..., 10, 15, 20
	$h_R$: Expansion hop count of replacement network	1, ..., 10, 15, 20
Interaction	Interaction rule	Pairwise game (PD), N-player game (PGG)
	*b*: Benefit of a game	PD:$\{1.1, 1.3, 1.5, 1.7, 1.9\}$, PGG:$\{2.0, 3.0, 4.0, 5.0, 6.0\}$
	*c*: Cost or contribution of a game	1.0
Replacement	Replacement rule	Birth–Death (BD), Death–Birth (DB), Imitation (IM)
	$\delta$: Strength of selection	$2^0, 2^{-1}, 2^{-2}, 2^{-3}, 2^{-4}, 0.01$
	$\mu$: Mutation rate	0.00, 0.01
Others	*N*: Population	1000
	*g*: Generations	10,000
	Trial count	100

## Results

**Figure 2:**
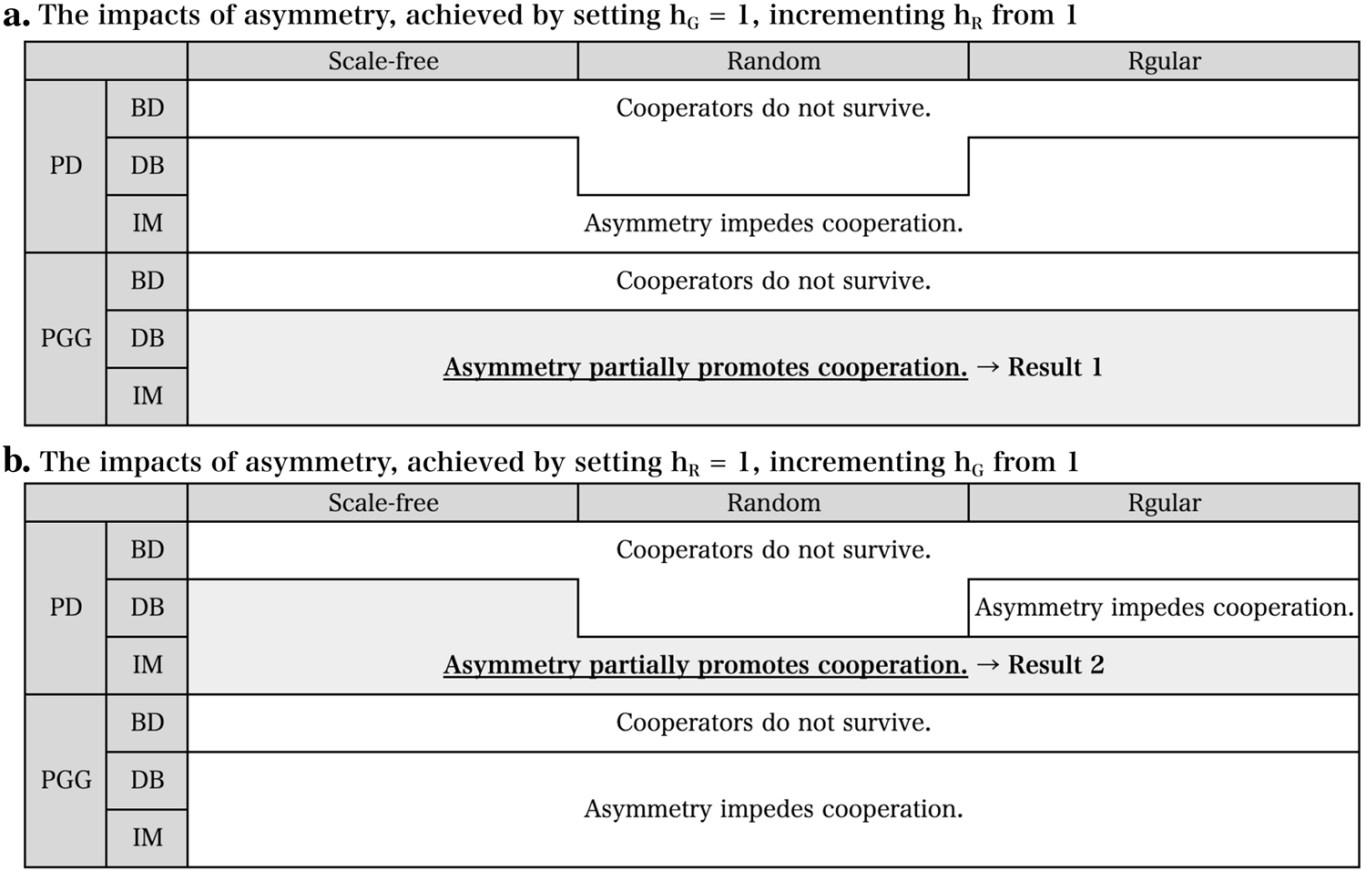
Overview of results for combinations of two game rules (PD and PGG), three strategy replacement rules (BD, DB, and IM), and three types of base network structures (Scale-free, Random, and Regular).

We examine the relationship between symmetries in the scope of interaction and strategy replacement and the evolution of cooperation through simulations. By ”symmetry”, we mean that the expansion hop count of the interaction network ($h_G$) and the count of the replacement network ($h_R$) are similar, while ”asymmetry” refers to the opposite case where $h_G$ and $h_R$ are dissimilar. As depicted in Fig. 2, the simulation results align with the findings of previous studies in many cases. However, in two specific regions, trends emerged that clearly diverged from previous studies, demonstrating that asymmetry can partially promote cooperation, or at the very least, not impede it.

**Figure 3:**
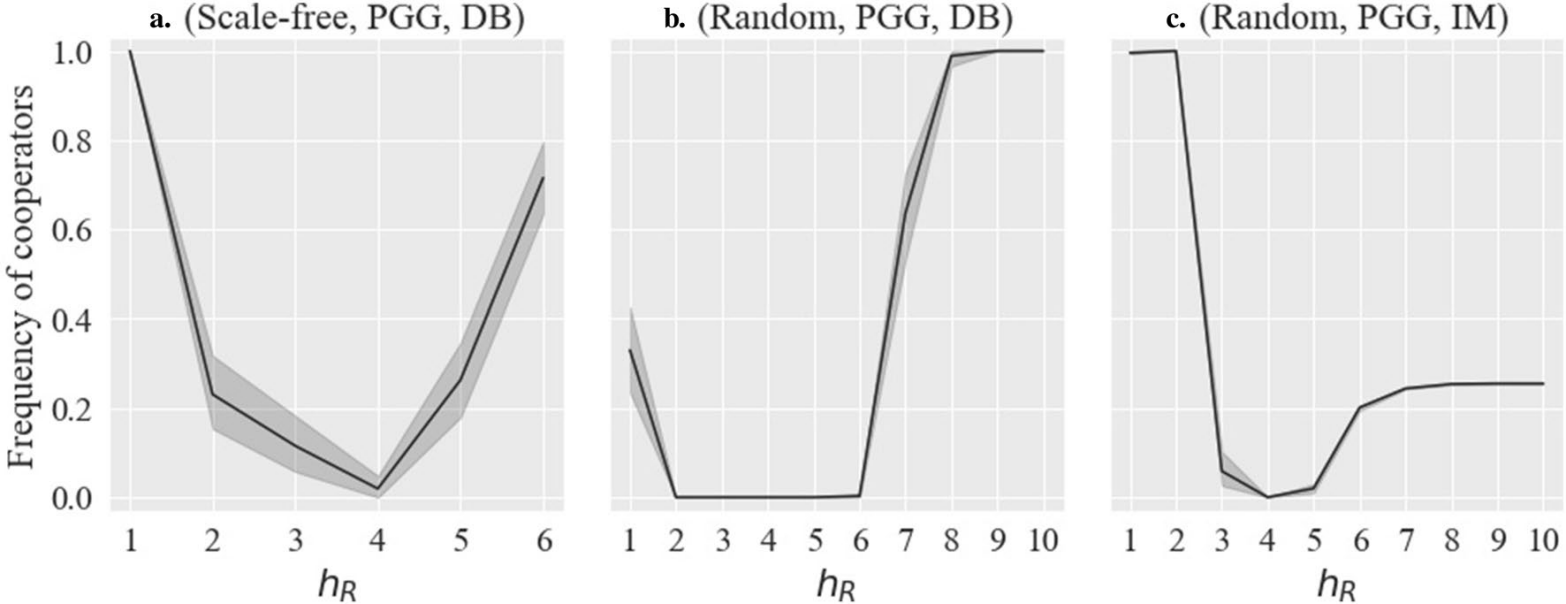
Example of results 1: ${h_G = 1, }\, b = 5.0,\, \delta = 1.0,\, \mu = 0.0$.

**Figure 4:**
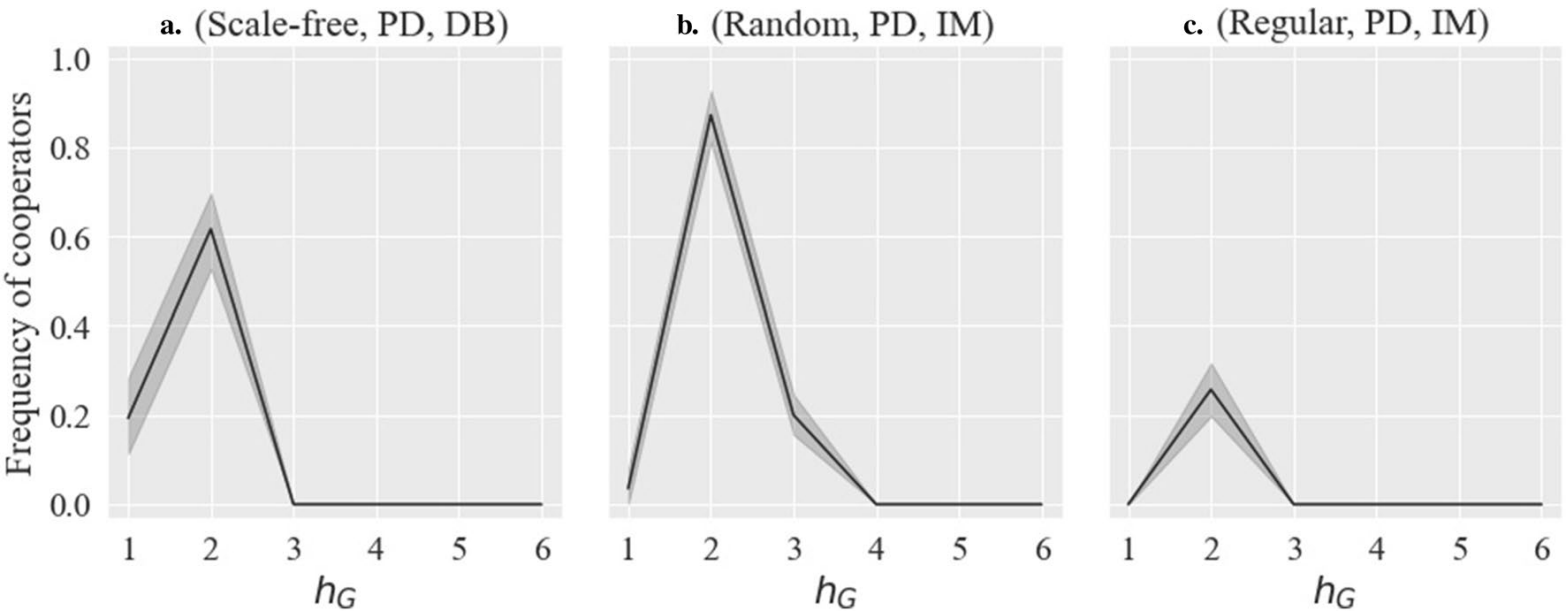
Example of results 2: ${h_R = 1, }\, b = 1.1,\, \delta = 0.01,\, \mu = 0.0$.

1. As Fig. 3 shows some typical examples, in the cases of (All base network types, PGG, DB or IM), when $h_G$ is fixed at 1 and $h_R$ increases, the cooperation rate initially decreases, consistent with previous studies^15,16,18,22^. However, as the asymmetry further increases, the cooperation rate unexpectedly recovers or, at the very least, ceases to decline. This phenomenon is unique to our study and has not been mentioned in previous studies. When $h_G$ is any value other than 1, cooperation does not spread at all and is therefore fixed at 0. Indeed, when comparing $h_R=1$ and $h_R=6$ and above in Fig. 3a and c, it might seem that asymmetry does not increase the cooperation rate since the cooperation rate is lower at the latter than at the former. However, the occurrence of a rising line with higher cooperation rates as asymmetry increases, even if only to a partial extent, is an important exception to previous studies.
2. As Fig. 4 shows some typical examples, in the cases of (Scale-free, PD, DB) and (All base network types, PD, IM), when $h_R$ is fixed at 1 and $h_G$ increases from 1, the cooperation rate rapidly increases at $h_G=2$ and then falls rapidly. This is another unique phenomenon observed in our study. When $h_R$ is other than 1, asymmetry generally inhibits cooperation, as concluded in previous studies (see SI.1).

The main finding of the above results is that the observed graphs exhibit a partially increasing trend, in contrast to the monotonic decrease reported in previous studies. Variations in *b*, $\mu$, and $\delta$ generally do not significantly influence the qualitative trends; therefore, our focus is primarily on game rules, strategy replacement rules, base network structure, $h_G$, and $h_R$. However, as an exception in Result 2, (Scale-free or Random, PD, IM), spikes occur when $\delta$ and *b* are small; in other cases, asymmetry inhibits cooperation. Only graphs related to our unique results are shown above. The other results confirm the robustness of previous studies or are not clearly different from them. Graphs of the results for all parameter patterns are available in the Supplementary Information.

### Mechanism

**Figure 5:**
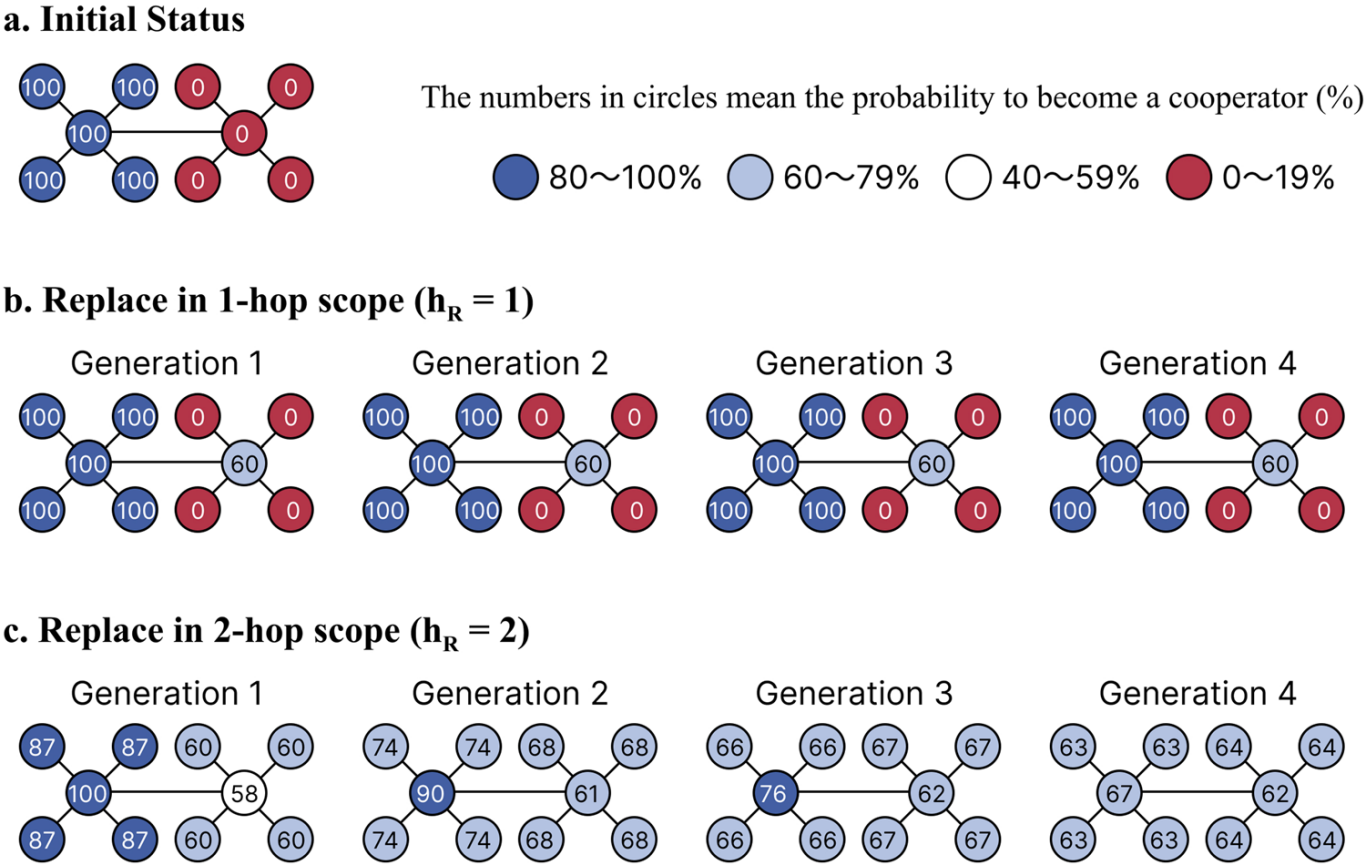
A simplified model illustrating the partially increasing trend of cooperation rate. (**a**) Initial state of the model. It is assumed that all agents in the left star are cooperators, while all agents in the right star are defectors. The number in each circle indicates the probability of each agent becoming a cooperator in each state. Interactions occur within a 1-hop range in both (**b**) and (**c**). Strategy replacements occur within a 1-hop range in (**b**) and a 2-hop range in (**c**). (**b**) represents symmetrical communication, while (**c**) denotes asymmetrical communication.

To illustrate what is happening in each generation and to investigate the emergence of the climbing part of Result 1, we consider a small and simple network, as shown in Fig. 5a. This network is a simplification of the nature of scale-free networks, focusing on the property that many nodes have only a few edges while a few nodes have many edges. It comprises two small star networks, one on each side, with each star consisting of a hub and four surrounding leaves. Initially, all members on the left star are assumed to be cooperators, and all members on the right star are assumed to be defectors. We simulate interactions (PGGs) and strategy replacements implemented on this network. We simplify not only the network structure but also simplify the rules as follows: (1) All agents are sequentially selected as the focal agent and execute interactions. (2) The focal agent randomly selects at most three agents directly connected to it to play a PGG. The scope of interactions is fixed at 1 hop ($h_G = 1$). (3) In each game, the cost contributed by a cooperator is 1 ($c = 1$), and the resources gathered are multiplied by 2 ($b/c = 2$) and distributed equally among the participants, including both cooperators and defectors. (4) All agents are sequentially selected as the focal agent and execute replacements. (5) The focal agent (A) randomly selects one agent (B) at a distance of $h_R$ hops ($h_R = 1 \ or \ 2$) from A, and if B’s payoff is higher than that of A, A imitates B’s strategy; otherwise, A does nothing.

Simulations of this model indicate that a two-hop strategy replacement ($h_R=2$) results in higher cooperation than a one-hop replacement ($h_R=1$) after approximately four generations (Fig. 5b, c). This result is consistent with the climbing part of Result 1, which shows that cooperation improves as the scope of replacement expands. In the first generation, we observe a significant impact of increasing $h_R$ on the cooperation rate of the leaves, but we see little effect on the hubs. After the second generation, the probability of each agent becoming a cooperator remains relatively stable when $h_R = 1$. However, when $h_R = 2$, the differences between stars and between hubs and leaves gradually disappear, and all nodes become cooperators with a probability of approximately 62–67%. This is because at $h_R = 1$, the leaves are influenced only by the hubs in their own star to which they are directly connected. Conversely, at $h_R = 2$, they are more susceptible to the hubs in another star. As a result, each agent has an equal chance of becoming a cooperator, not just equalized to the average value of $h_R = 1$ (56%), but with all members having a 62–67% chance of becoming a cooperator. Increasing $h_R$ significantly raises the cooperation rate of the leaves on D-star, decreases the cooperation rate of C-star, and leaves the cooperation rate of the hub on D-star unchanged. Comparing these effects, we find that the impact of increased cooperation among the leaves on D-star is more significant, resulting in a higher average cooperation rate. In summary, increasing $h_R$ eliminates the force that promotes cooperation originating from the hub but enhances the ability to learn from distant partners and promotes cooperation among leaves that would otherwise be defectors in a narrower scope of replacement. The latter effect can be more substantial, leading to an overall increase in the cooperation rate in some cases. This result is valid for the mini model. We cannot assert that Result 1 is completely generated by the same mechanism, but we present it as an initial hypothesis.

## Discussion

We found in this study that asymmetries in the scope of interactions and strategy replacements can promote cooperation in certain cases. Previous studies^15,16,18,22^ have indicated that symmetrically conducting interactions and replacements with the same partner as much as possible promotes cooperation, but these conclusions were based on networks that corresponded to respective research purposes rather than on scope-focused multiplex networks. As these studies were conducted under different assumptions, the fact that the conclusions differ between the previous studies and ours does not by itself immediately lead us to consider this a surprising finding. However, previous studies, conducted under a variety of assumptions, consistently state that symmetry promotes cooperation, and few cases contrary to this conclusion are known. Hence, importantly, even under different conditions, we obtained different conclusions and identified a network structure that produced them. In this context, we performed extensive simulations of various network structures, interaction rules, and replacement rules, and we found that in the following two cases, asymmetry promotes cooperation, contrary to previous studies. (1) In the case of public goods games on scale-free networks, when the scope of interactions is narrow, replacements over a wide range are more likely to spread cooperation than over a medium range. (2) In the case of prisoner’s dilemma games on scale-free, random, and regular networks, when the scope of replacements is narrow, only when the scope of interactions is 2 hops are there cases where the cooperation rate increases sharply. These are novel phenomena that have not been mentioned before and might contribute to a deeper understanding of the relationship between the evolution of cooperation and the symmetry of communication.

The difference between our study and prior studies lies in the network structure used and whether or not fixation probability under conditions of weak selection is used. While prior studies measured symmetry by using the ratio of overlapping edges in the two layers, we assessed symmetry based on the scope of interactions and replacement. Since the former includes the latter, our study serves as a counterexample to previous studies. For (1), we presented a hypothesis regarding the mechanism; however, further studies are needed to determine whether this hypothesis holds for more general cases. Regarding (2), the mechanism remains unknown. Thus, future research is required to investigate the detailed mechanism and the conditions under which these phenomena occur.

Translating our conclusions to the real-world context, our findings suggest that when attempting to promote cooperation within a group, not only symmetric learning from the same partners involved in the interactions but also asymmetric learning from a broader range of partners beyond the scope of these interactions are possible. However, the model employed in this study is a theoretical abstraction, meaning that any derived conclusions are primarily theoretical. Applying this model to interpret what has actually transpired or to predict what may occur in reality might not be straightforward. Furthermore, while our model contributes to a deeper understanding of cooperative behavior, which is a foundational aspect of social activities, its direct application to social issues remains a challenge. For future research, it may be beneficial to refine our model to account for the complexity of human incentive systems. This may involve incorporating elements such as reputation^10,34–38^ and punishment^38–44^. Additionally, establishing connections with empirical studies in diverse fields such as economics, sociology, and anthropology may further enhance the model’s applicability and effectiveness.

## Supplementary Information

Supplementary Information.

## Data Availability

All data, as well as the code required to run the simulations and produce the plots, are provided at https://github.com/mas178/inaba2023a.
