# Supplementary material for: Evolution of cooperation in multiplex networks through asymmetry between interaction and replacement
Source: Sci Rep. 2023 Jun 17;13:9814. doi: 10.1038/s41598-023-37074-4 (PMC10276876; doi:10.1038/s41598-023-37074-4)
Supplement: Supplementary file 1 — Supplementary Information. [file 41598_2023_37074_MOESM1_ESM.pdf]

Evolution of cooperation in multiplex networks through asymmetry between interaction and replacement
Supplemental Information

Contents

1 Results of all simulations **1**
- 1.1 Pairwise game (prisoner's dilemma game: PD) . . . 1
  - 1.1.1 Scale-free network, PD, DB . . . 1
  - 1.1.2 Scale-free network, PD, IM . . . 3
  - 1.1.3 Random network, PD, DB . . . 5
  - 1.1.4 Random network, PD, IM . . . 7
  - 1.1.5 Regular network, PD, DB . . . 9
  - 1.1.6 Regular network, PD, IM . . . 11
  - 1.1.7 All networks, PD, BD . . . 12
- 1.2 N-player game (Public Goods Game: PGG) . . . 13
  - 1.2.1 Scale-free network, PGG, BD . . . 13
  - 1.2.2 Scale-free network, PGG, DB . . . 14
  - 1.2.3 Scale-free network, PGG, IM . . . 15
  - 1.2.4 Random network, PGG, BD . . . 16
  - 1.2.5 Random network, PGG, DB . . . 17
  - 1.2.6 Random network, PGG, IM . . . 18
  - 1.2.7 Regular network, PGG, BD . . . 19
  - 1.2.8 Regular network, PGG, DB . . . 20
  - 1.2.9 Regular network, PGG, IM . . . 21

2 Expanded network features **22**
- 2.1 Features of expanded scale-free networks . . . 22
- 2.2 Features of expanded random networks . . . 23
- 2.3 Features of expanded regular networks . . . 24

1 Results of all simulations

1.1 Pairwise game (prisoner's dilemma game: PD)

1.1.1 Scale-free network, PD, DB

Figure 1 and Figure 2 represent the same results but from different perspectives on the x-axis. Figure 1 illustrates Result 2, which shows how the cooperation rate sharply increases at an extended hop count of 2 in the interaction network, followed by a sharp decline. Figure 2 illustrates that the expansion of the strategy replacement network inhibits cooperation.

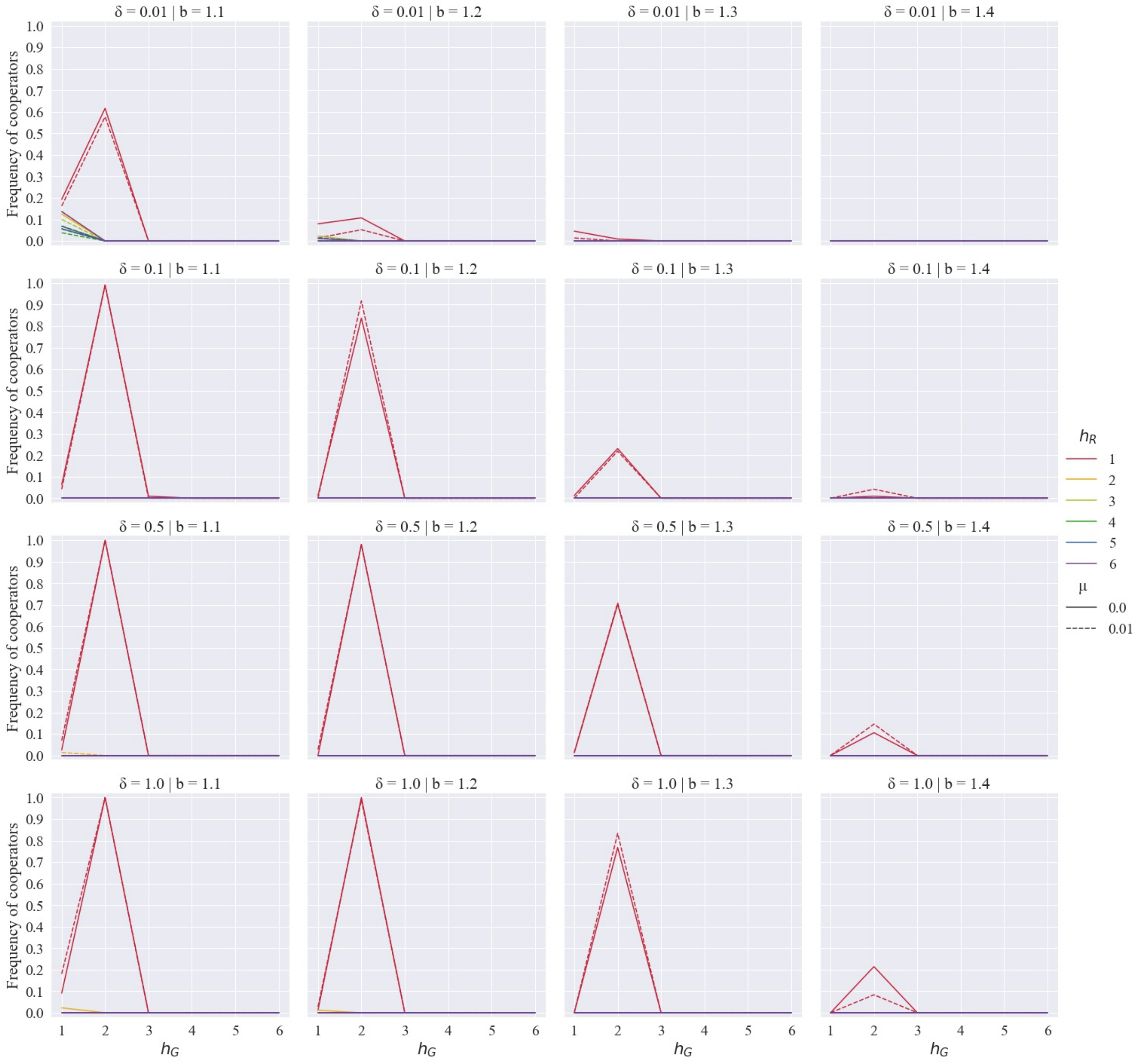

Figure 1: Scale-free, PD, DB (X-axis: h_G)

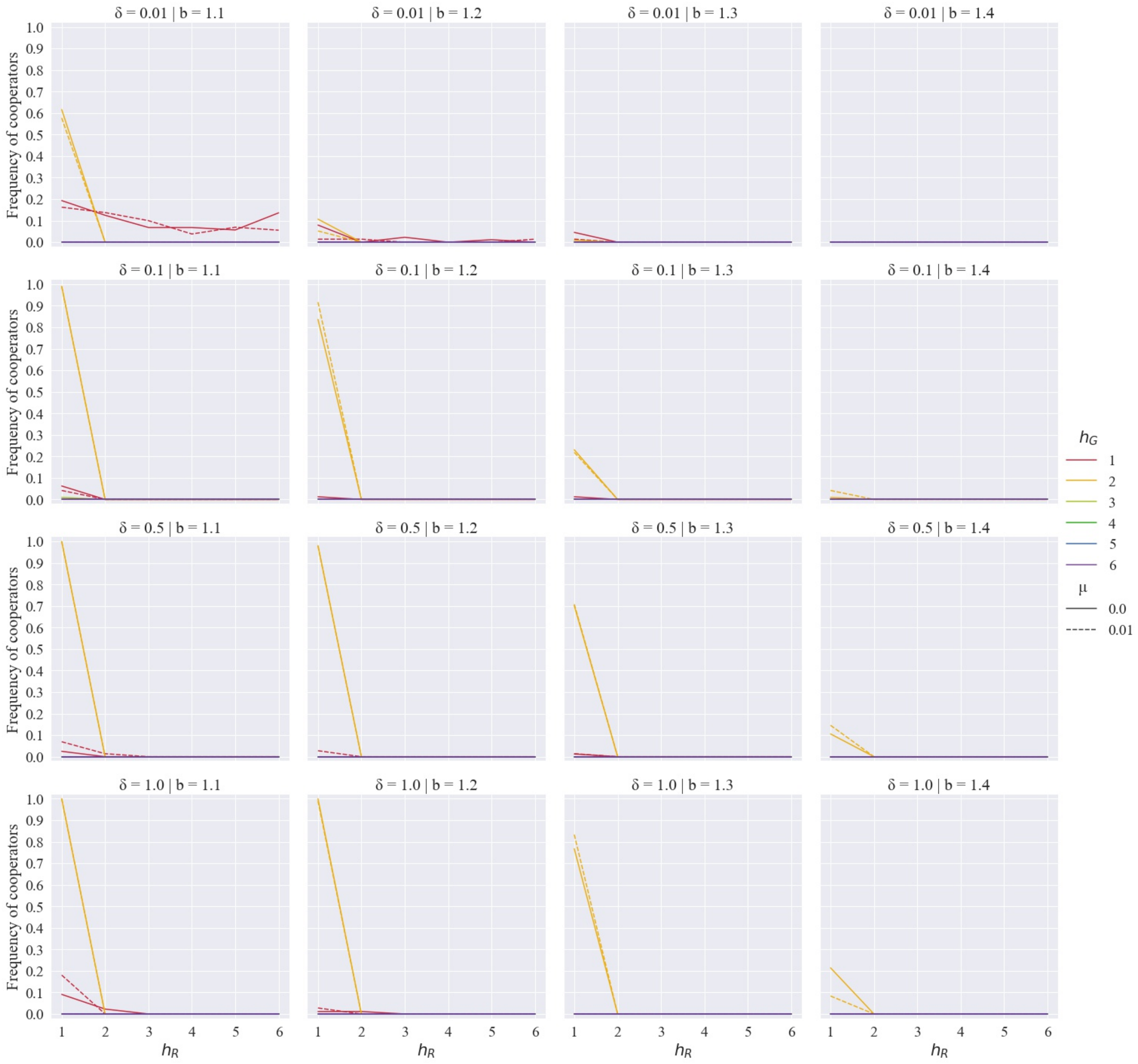

Figure 2: Scale-free, PD, DB (X-axis: h_R)

1.1.2 Scale-free network, PD, IM

Figure 3 and Figure 4 represent the same results but from different perspectives on the x-axis. Figure 3 partially illustrates Result 2, which shows how the cooperation rate sharply increases at an extended hop count of 2 in the interaction network, followed by a sharp decline. Most of Figure 3 and all of Figure 4 illustrate that the asymmetry in the scope between interactions and replacements inhibits cooperation.

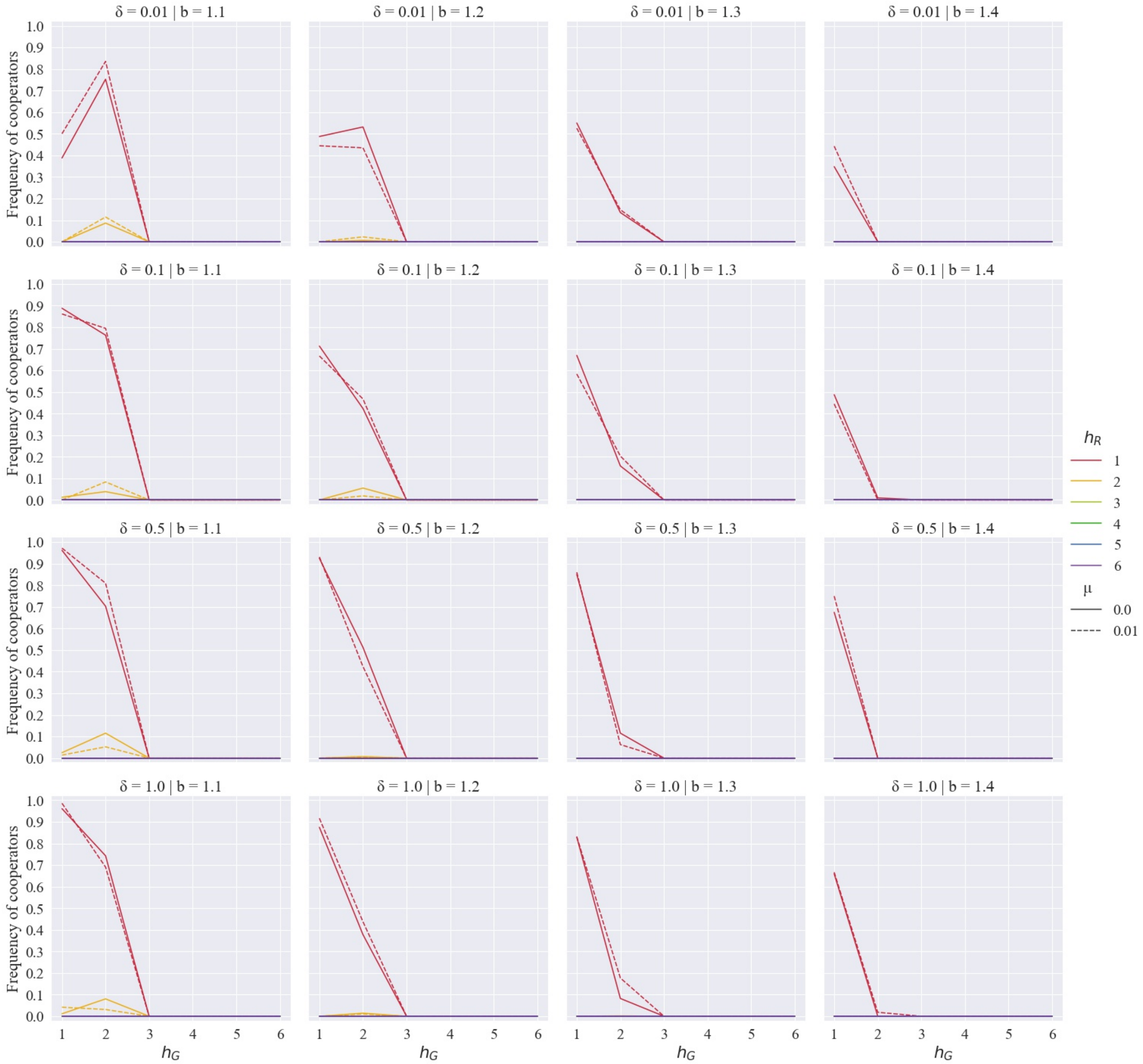

Figure 3: Scale-free, PD, IM (X-axis: h_G)

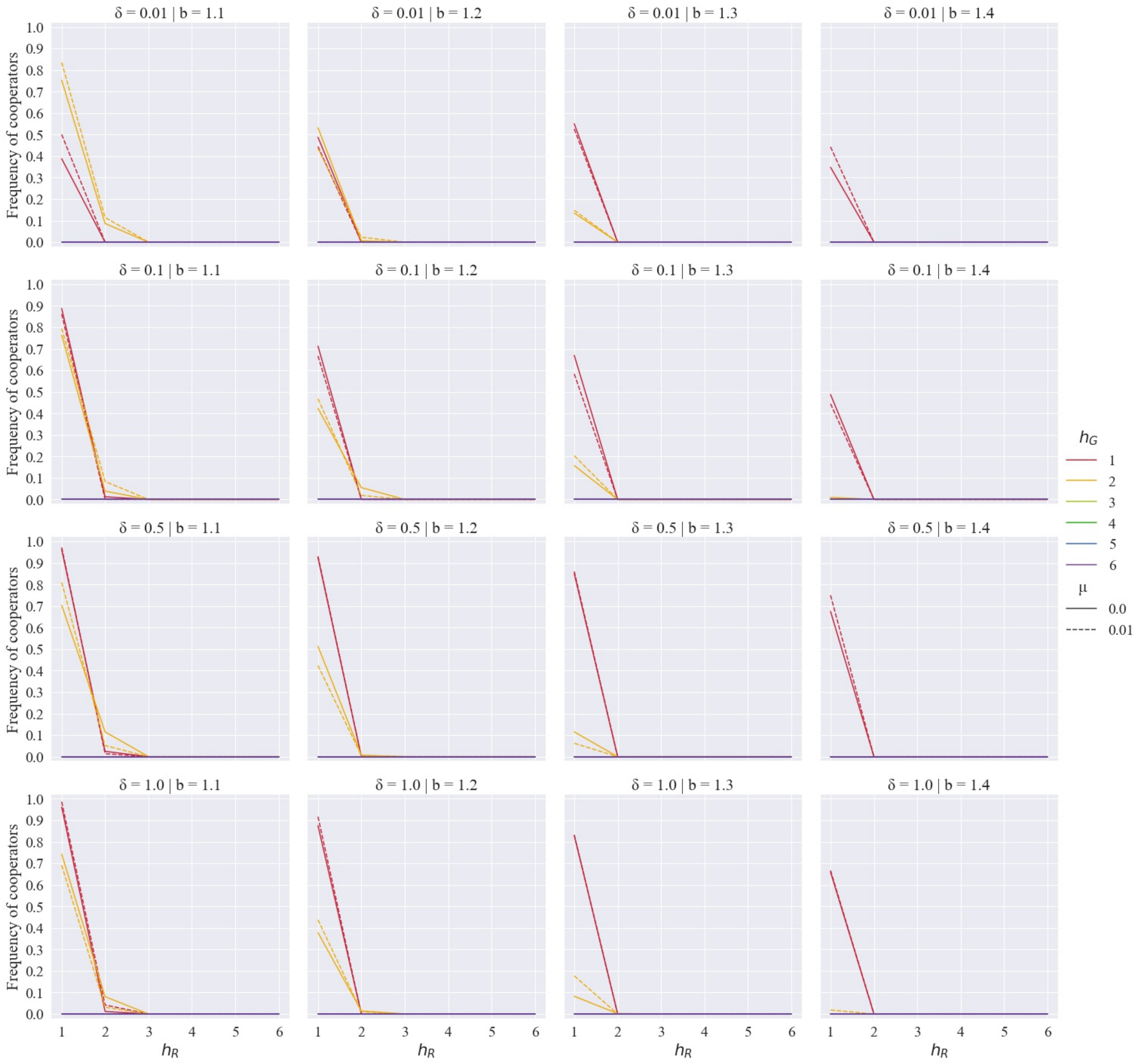

Figure 4: Scale-free, PD, IM (X-axis: h_R)

1.1.3 Random network, PD, DB

Figure 3 and Figure 4 represent the same results but from different perspectives on the x-axis. Cooperation rarely evolves under these conditions.

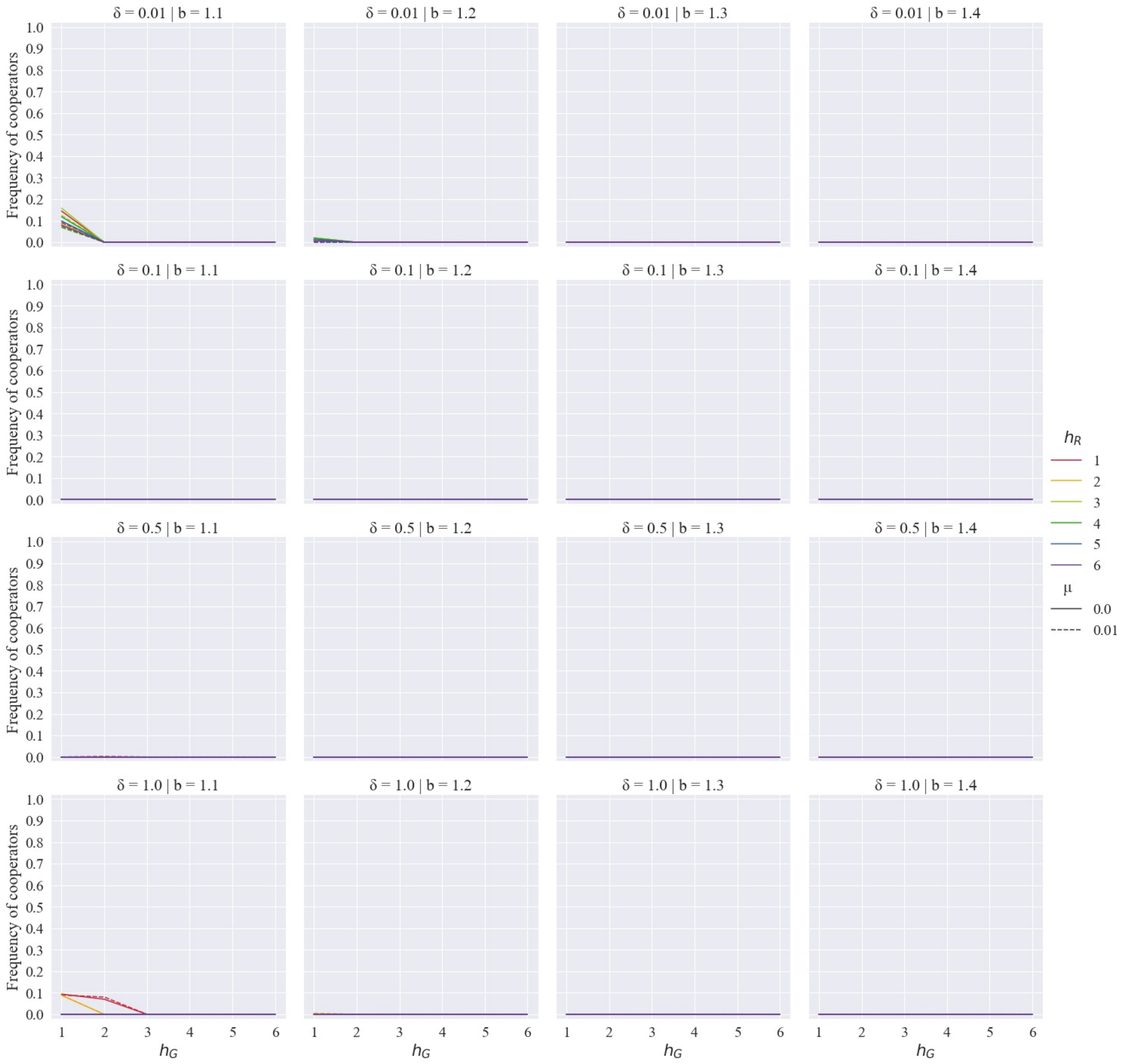

Figure 5: Random, PD, DB (X-axis: h_G)

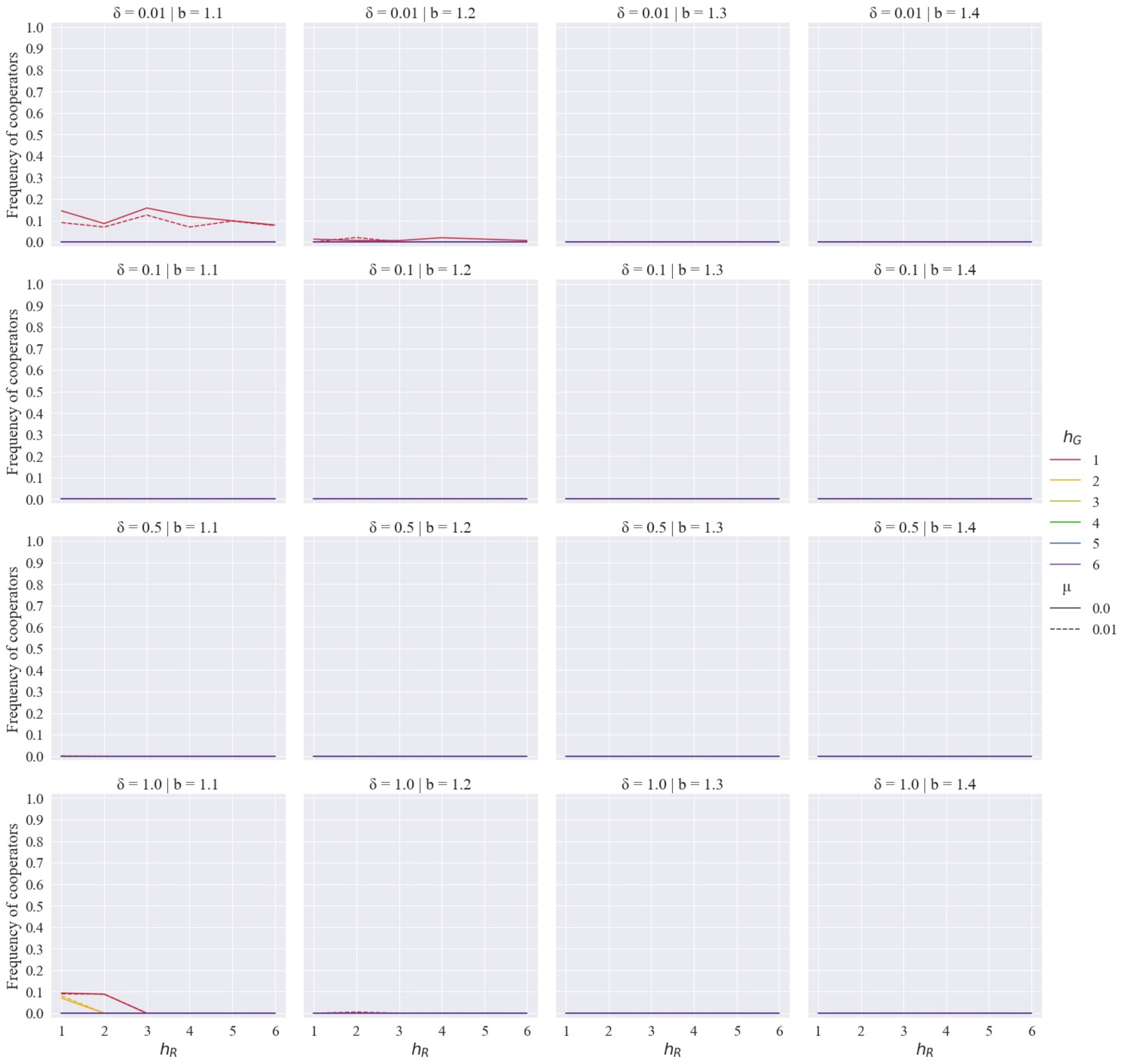

Figure 6: Random, PD, DB (X-axis: h_R)

1.1.4 Random network, PD, IM

Figure 7 and Figure 8 represent the same results but from different perspectives on the x-axis. Figure 7 illustrates Result 2, which shows how the cooperation rate sharply increases at an extended hop count of 2 in the interaction network, followed by a sharp decline. Figure 8 illustrates that the expansion of the strategy replacement network inhibits cooperation.

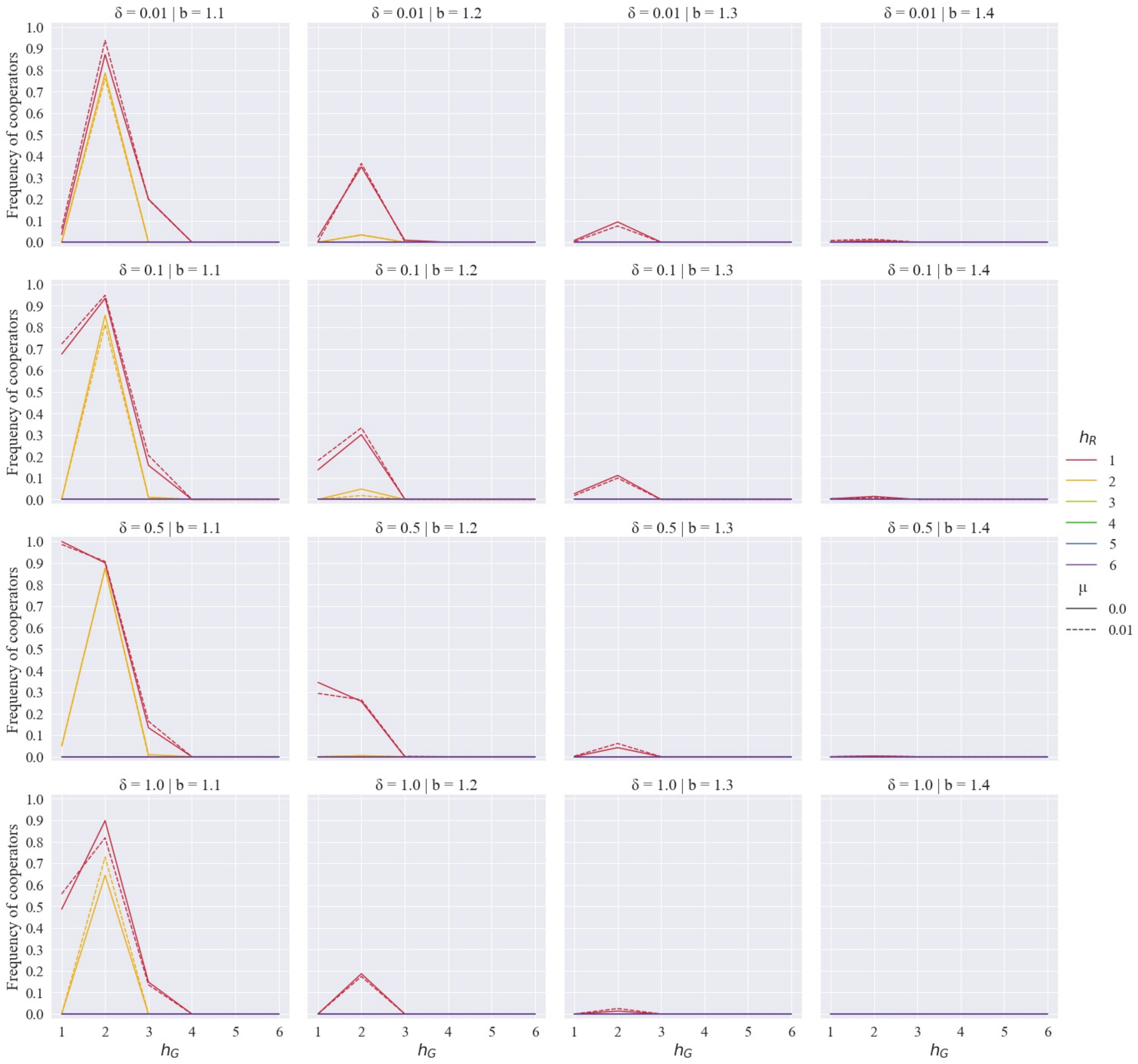

Figure 7: Random, PD, IM (X-axis: h_G)

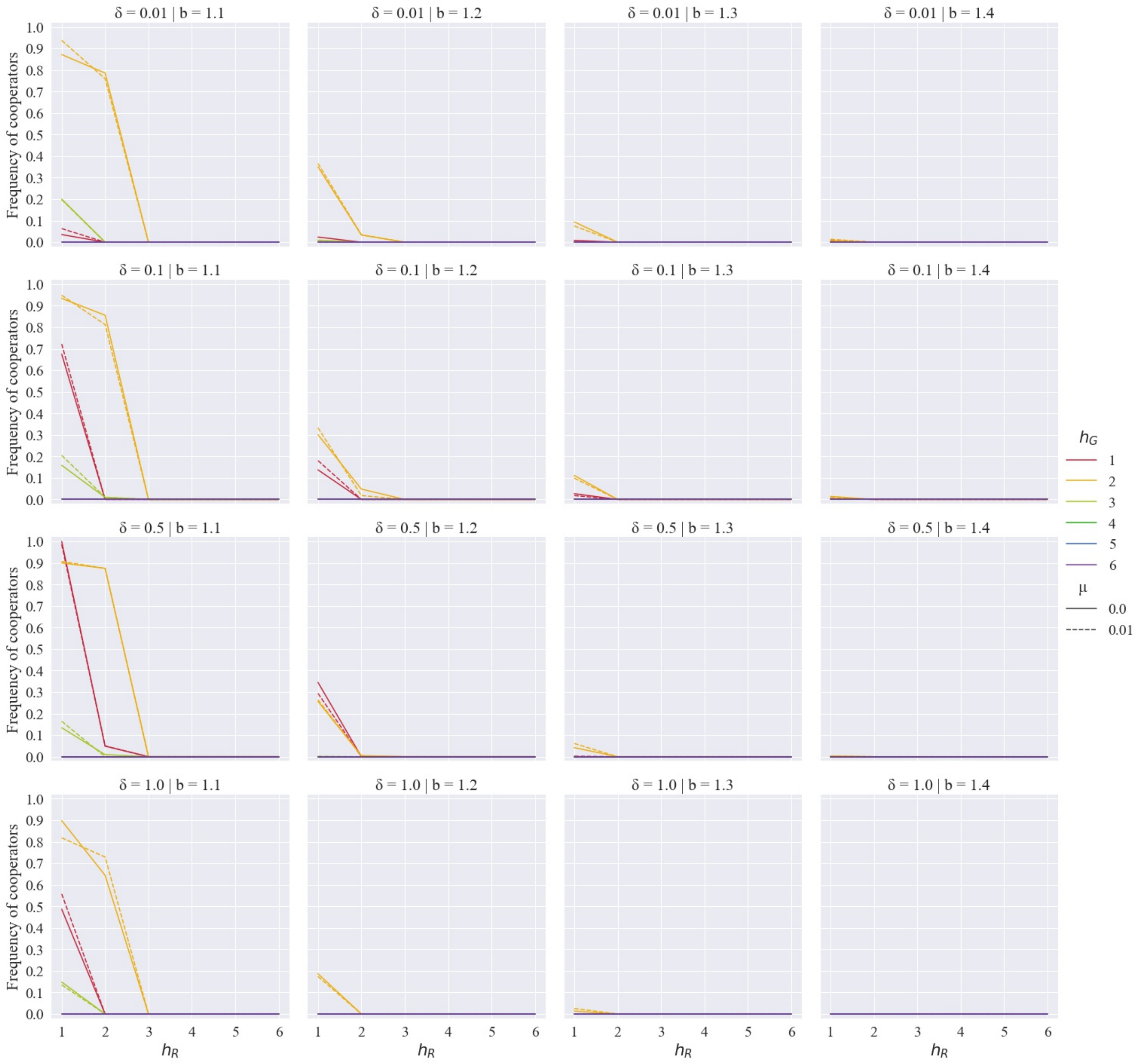

Figure 8: Random, PD, IM (X-axis: h_R)

1.1.5 Regular network, PD, DB

Figure 9 and Figure 10 represent the same results but from different perspectives on the x-axis. These figures illustrate that the asymmetry in the scope between interactions and replacements inhibits cooperation.

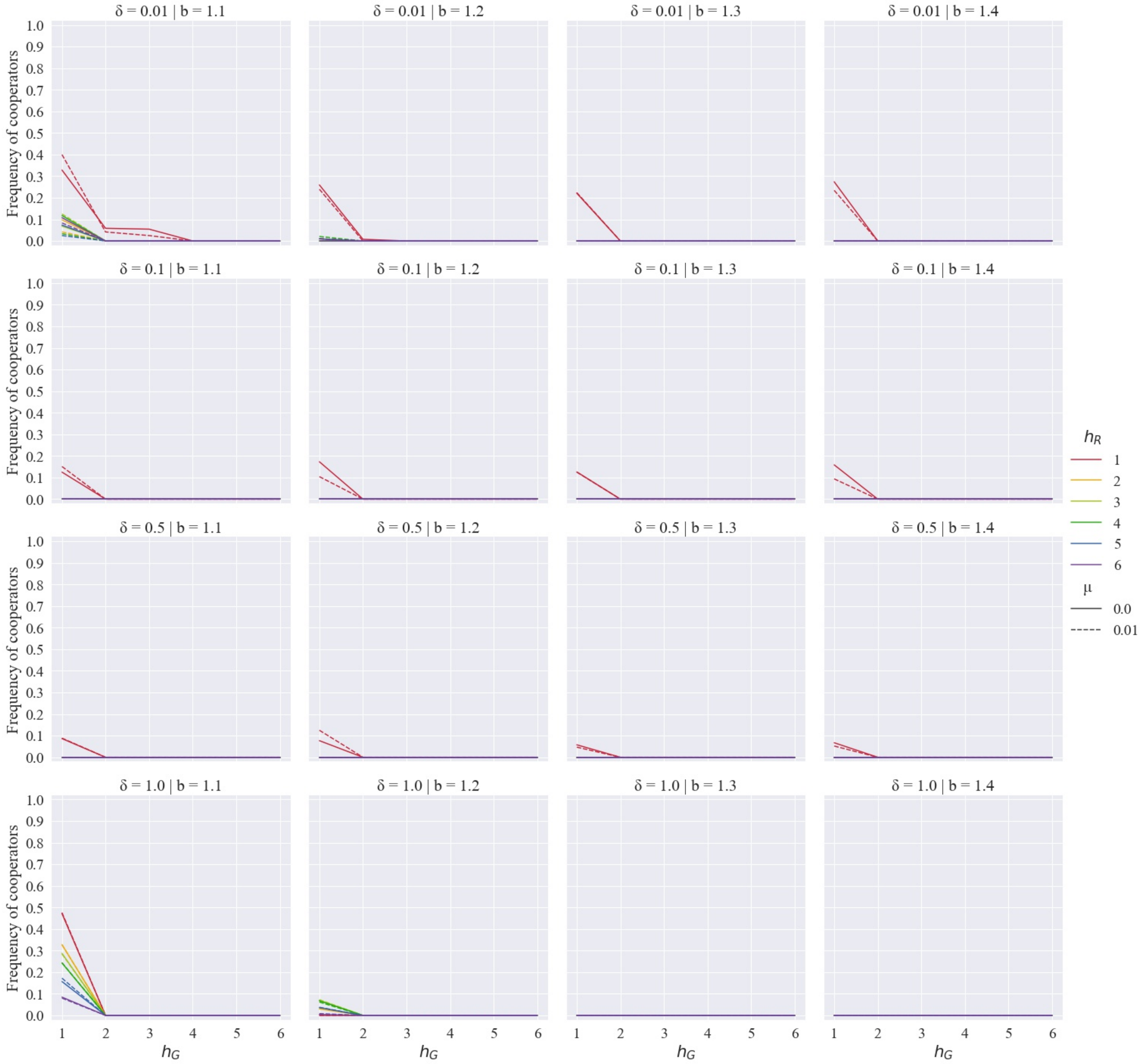

Figure 9: Regular, PD, DB (X-axis: h_G)

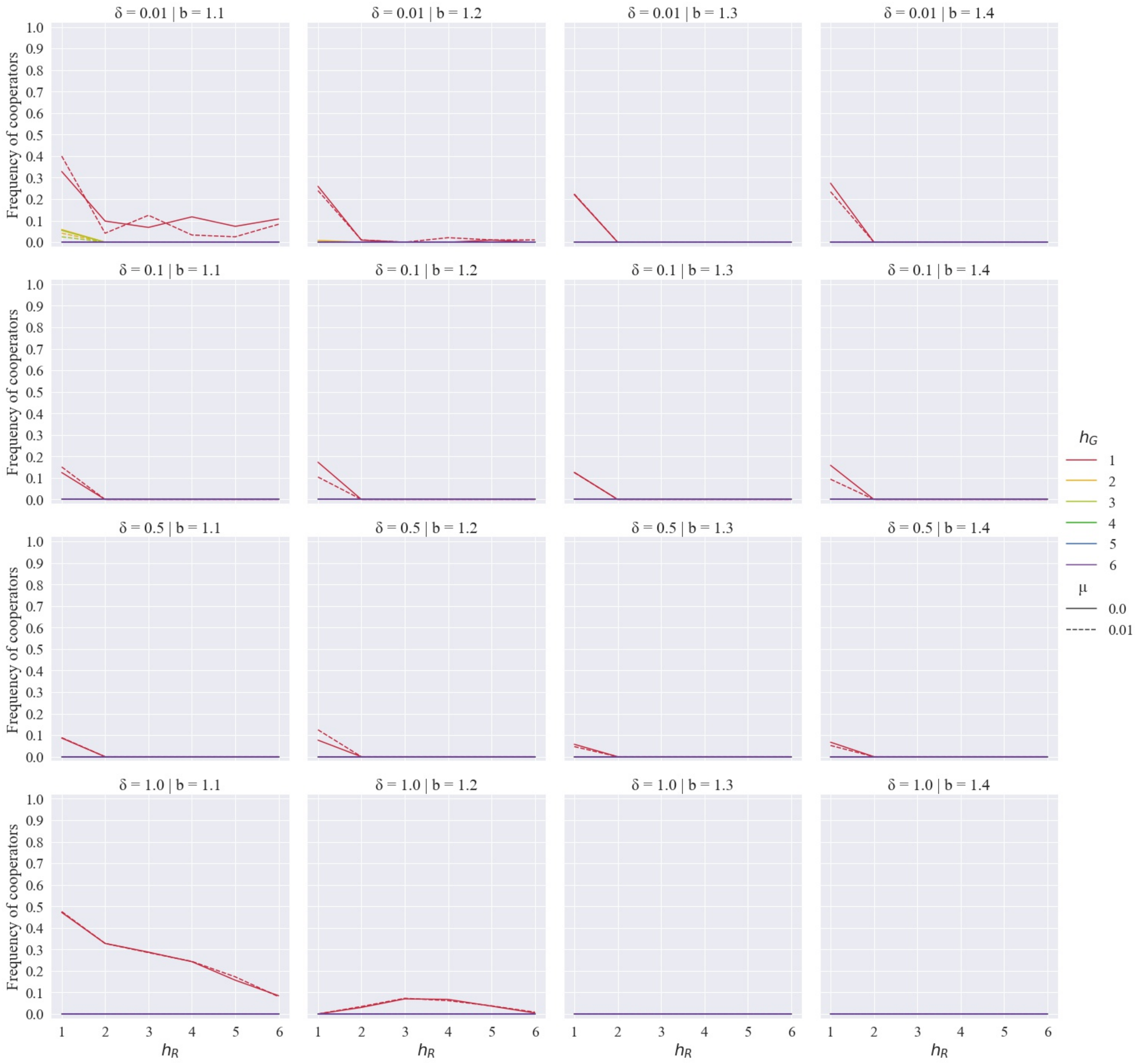

Figure 10: Regular, PD, DB (X-axis: h_R)

We have confirmed that the mountain shape at $\delta = 1.0$ and $b = 1.2$ is eliminated when the number of generations is increased from 10,000 to 100,000.

1.1.6 Regular network, PD, IM

Figure 11 and Figure 12 represent the same results but from different perspectives on the x-axis. Cooperation rarely evolves under these conditions, although Figure 11 partially illustrates Result 2, which shows how the cooperation rate sharply increases at an extended hop count of 2 in the interaction network, followed by a sharp decline.

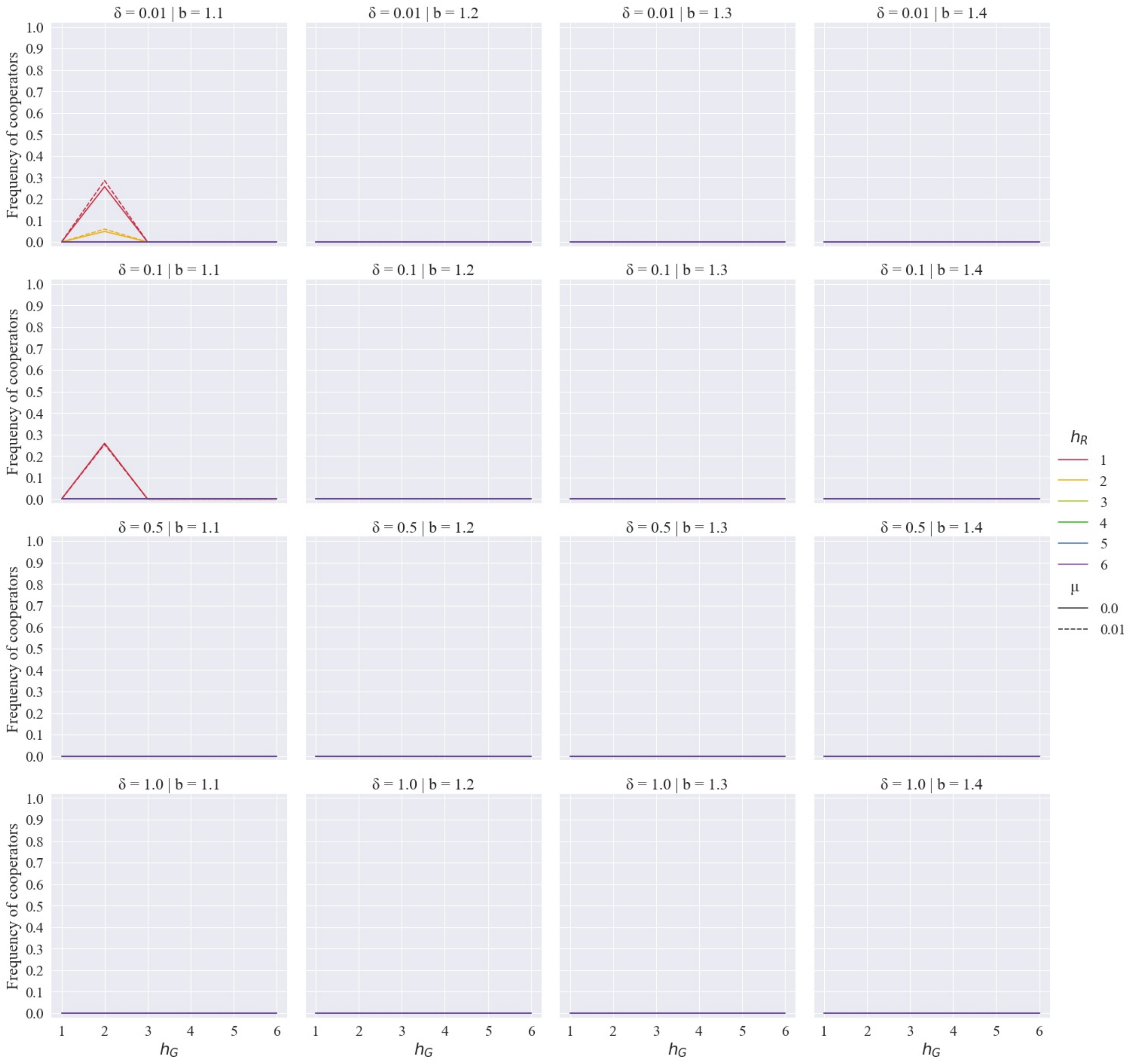

Figure 11: Regular, PD, IM (X-axis: h_G)

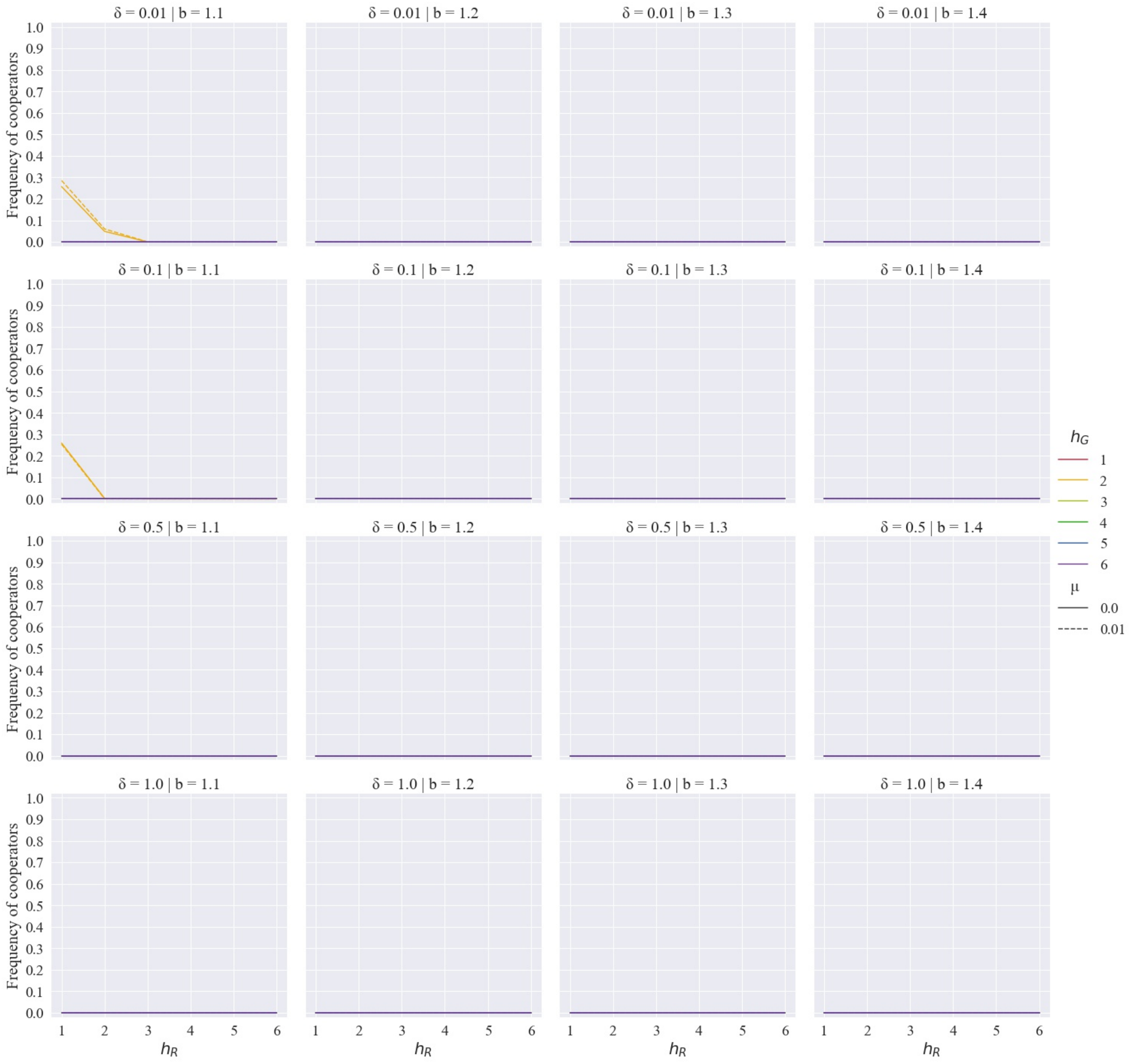

Figure 12: Regular, PD, IM (X-axis: h_R)

1.1.7 All networks, PD, BD

In the cases of PD and BD, cooperation does not evolve, regardless of the type of base network and the number of extended hops.

Table 1: Average cooperation rate and standard deviation in (all networks, PD, BD)

Base network	μ	σ
Scale-free	0.000171	0.000320
Random	0.000153	0.000302
Regular	0.000186	0.000388

1.2 N-player game (Public Goods Game: PGG)

1.2.1 Scale-free network, PGG, BD

Cooperation rarely evolves under these conditions.

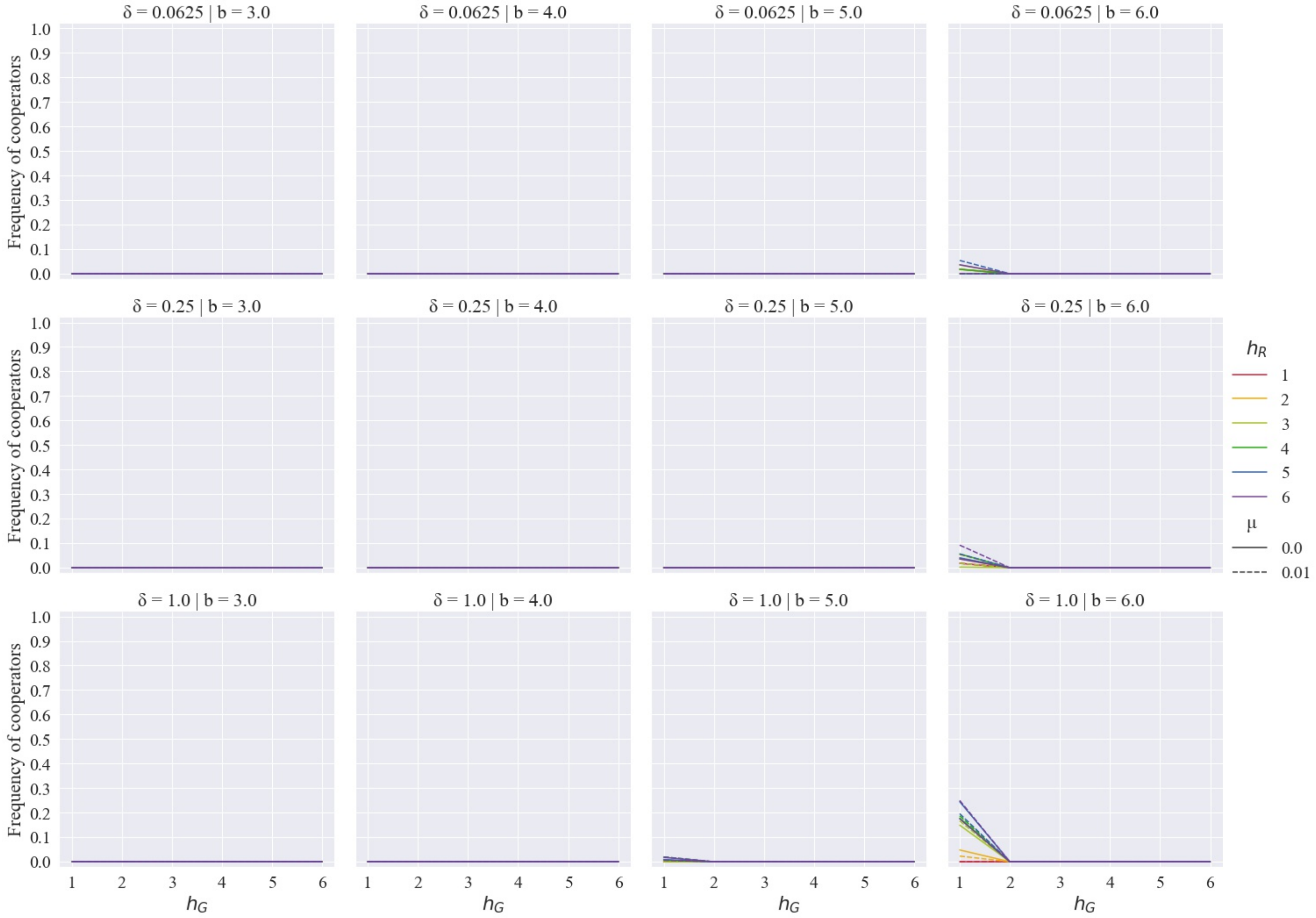

Figure 13: Scale-free, PGG, BD

1.2.2 Scale-free network, PGG, DB

Figure 14 and Figure 15 represent the same results but from different perspectives on the x-axis. Figure 14 illustrates that cooperation rarely evolves when the interaction scope is greater than 2. Figure 15 illustrates Result 1, which demonstrates how the cooperation rate first decreases and then increases with a larger replacement scope.

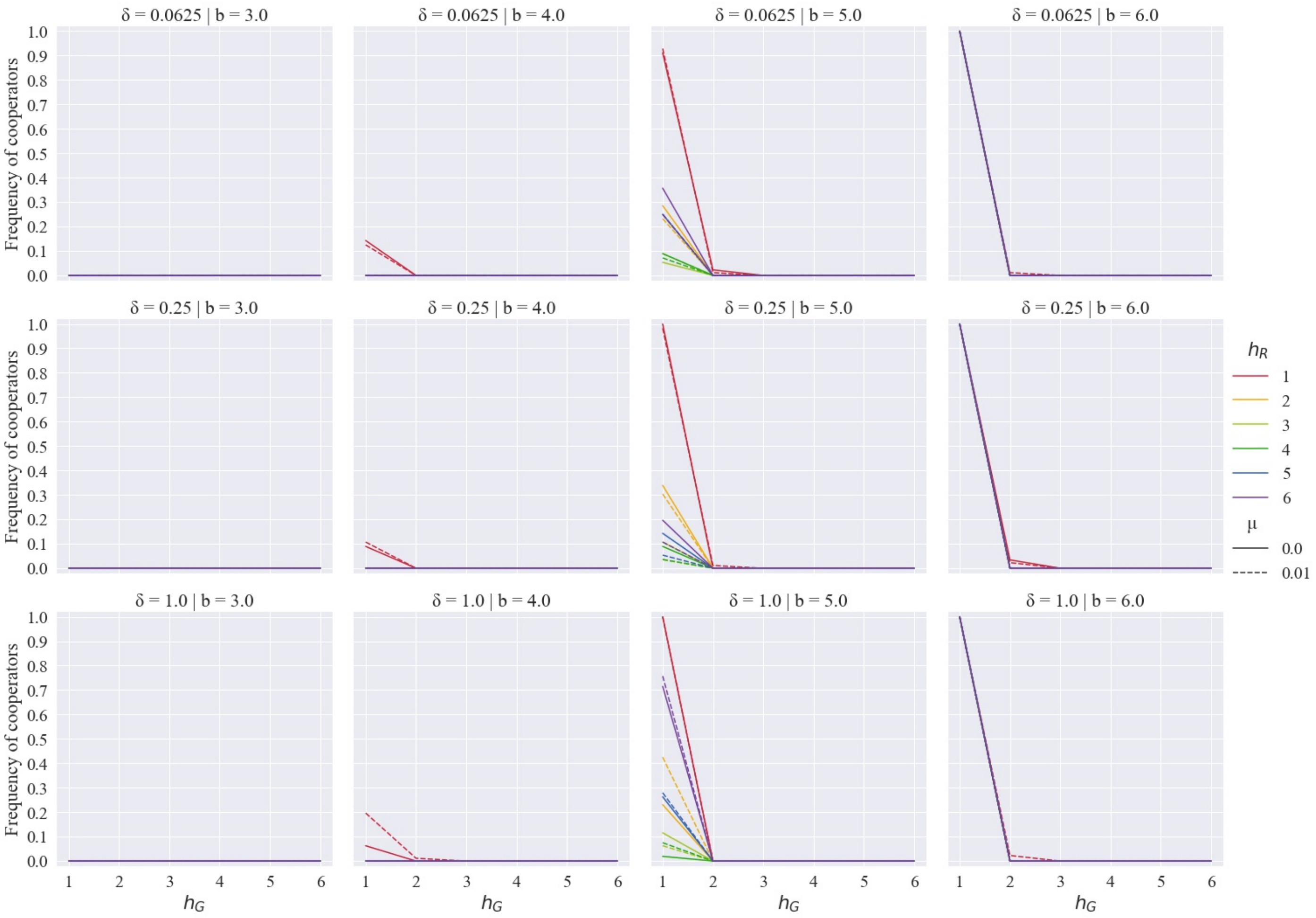

Figure 14: Scale-free, PGG, DB (X-axis: h_G)

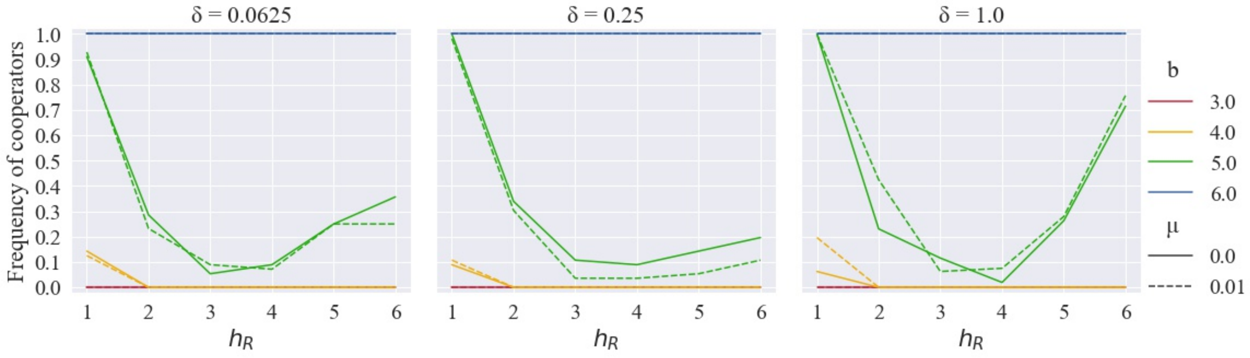

Figure 15: Scale-free, PGG, DB (X-axis: h_R, $h_G = 1$)

1.2.3 Scale-free network, PGG, IM

Figure 16 and Figure 17 represent the same results but from different perspectives on the x-axis. Both figures illustrate that the cooperation rate decreases with asymmetric communication scope, consistent with previous studies.

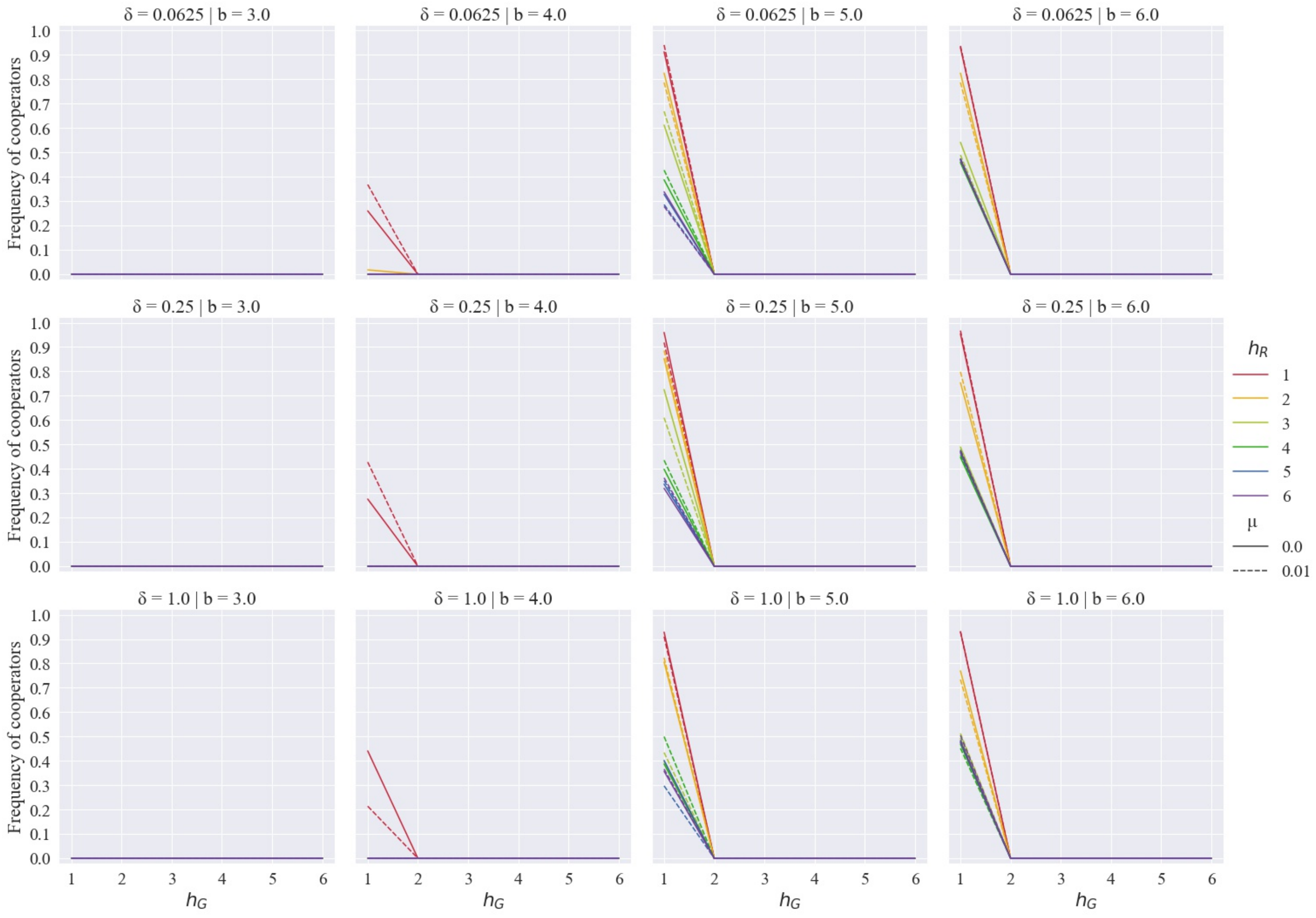

Figure 16: Scale-free, PGG, IM (X-axis: h_G)

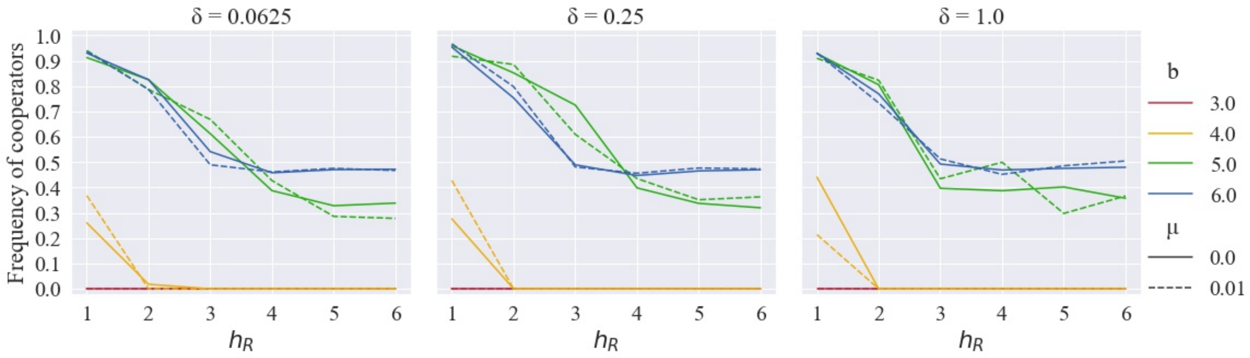

Figure 17: Scale-free, PGG, IM ($h_G = 1$, X-axis: h_R)

1.2.4 Random network, PGG, BD

Cooperation rarely evolves under these conditions.

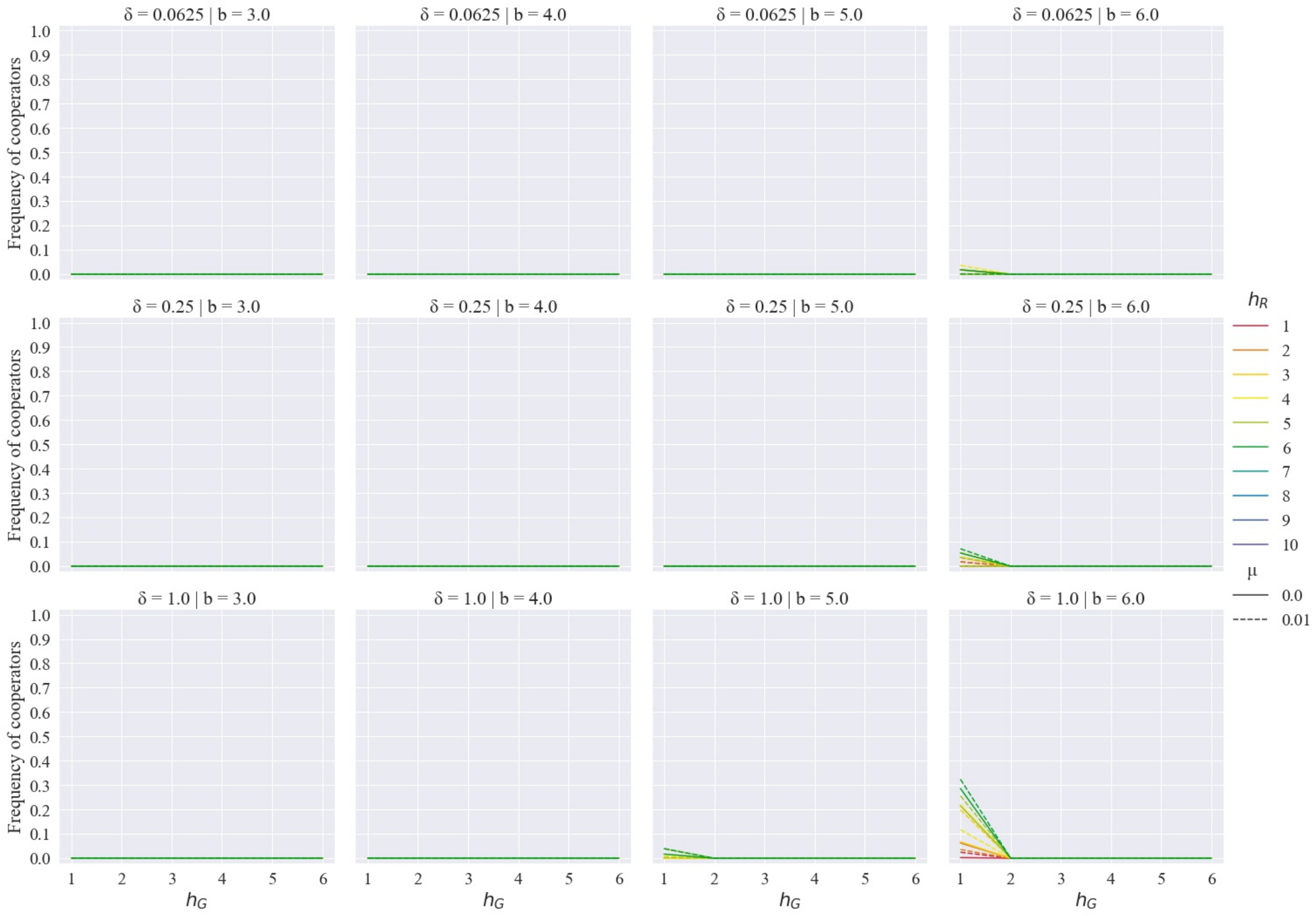

Figure 18: Random, PGG, BD

1.2.5 Random network, PGG, DB

Figure 14 and Figure 15 represent the same results but from different perspectives on the x-axis. Figure 14 illustrates that cooperation rarely evolves when the interaction scope is greater than 2. Figure 15 illustrates Result 1, which demonstrates how the cooperation rate first decreases and then increases with a larger replacement scope.

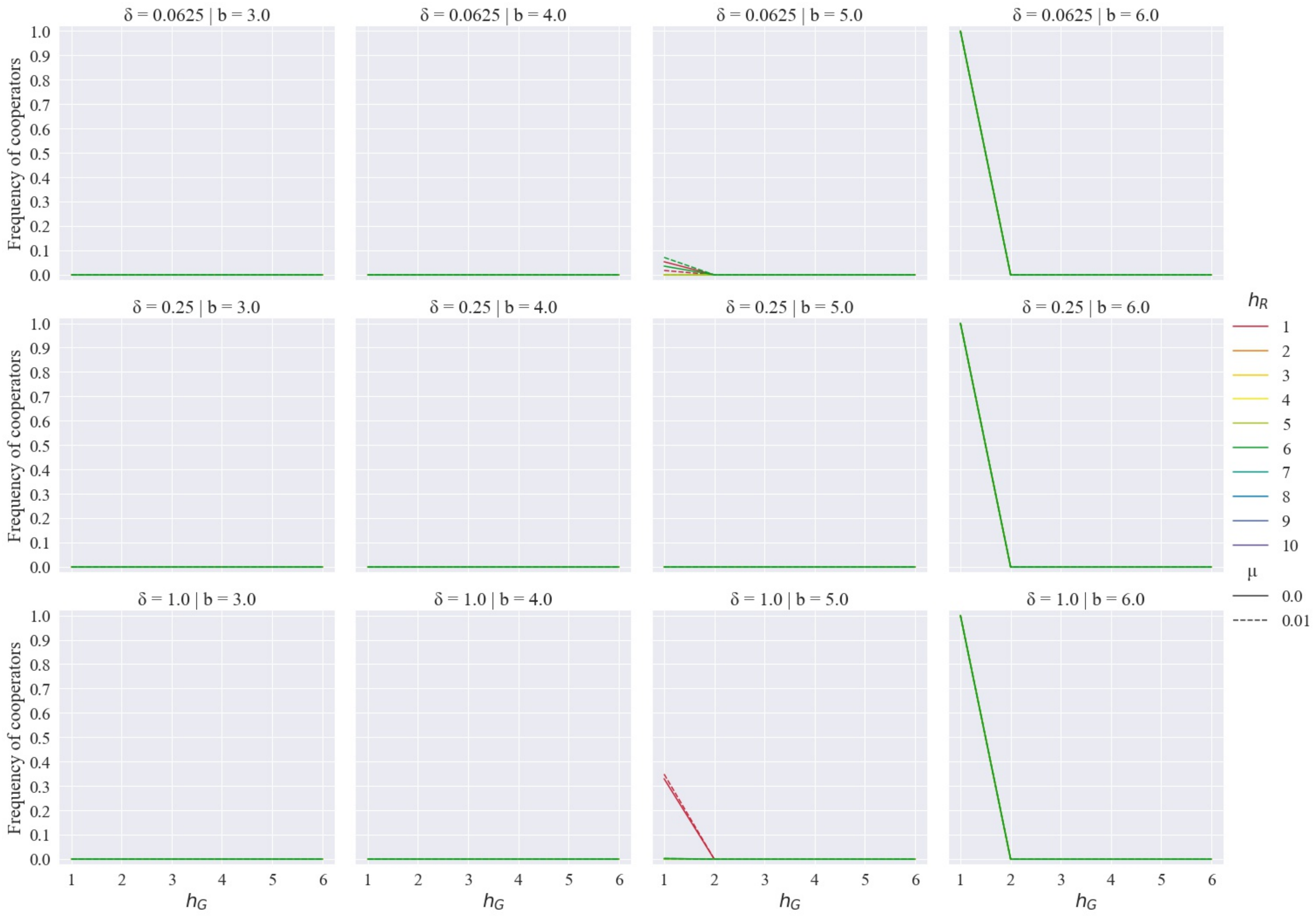

Figure 19: Random, PGG, DB (X-axis: h_G)

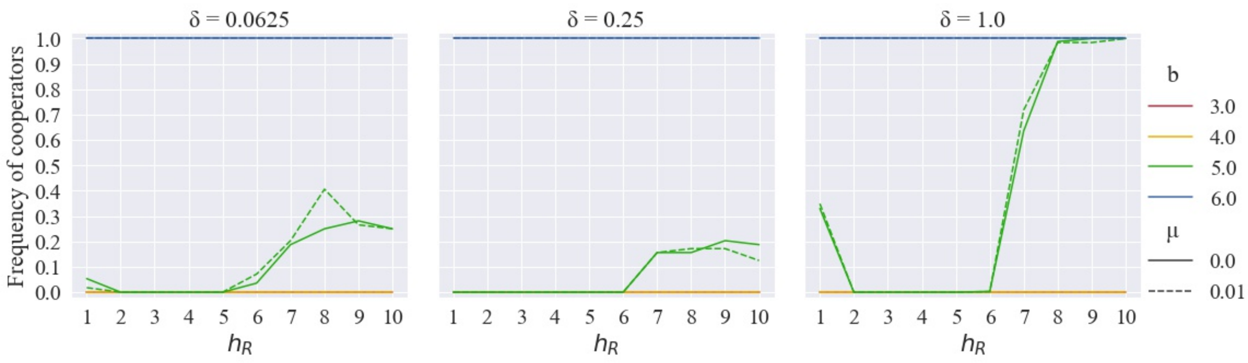

Figure 20: Random, PGG, DB ($h_G = 1$, X-axis: h_R)

1.2.6 Random network, PGG, IM

Figure 21 and Figure 22 represent the same results but from different perspectives on the x-axis. Figure 21 illustrates that cooperation rarely evolves when the interaction scope is greater than 2. Figure 22 illustrates Result 1, which demonstrates how the cooperation rate first decreases and then increases with a larger replacement scope.

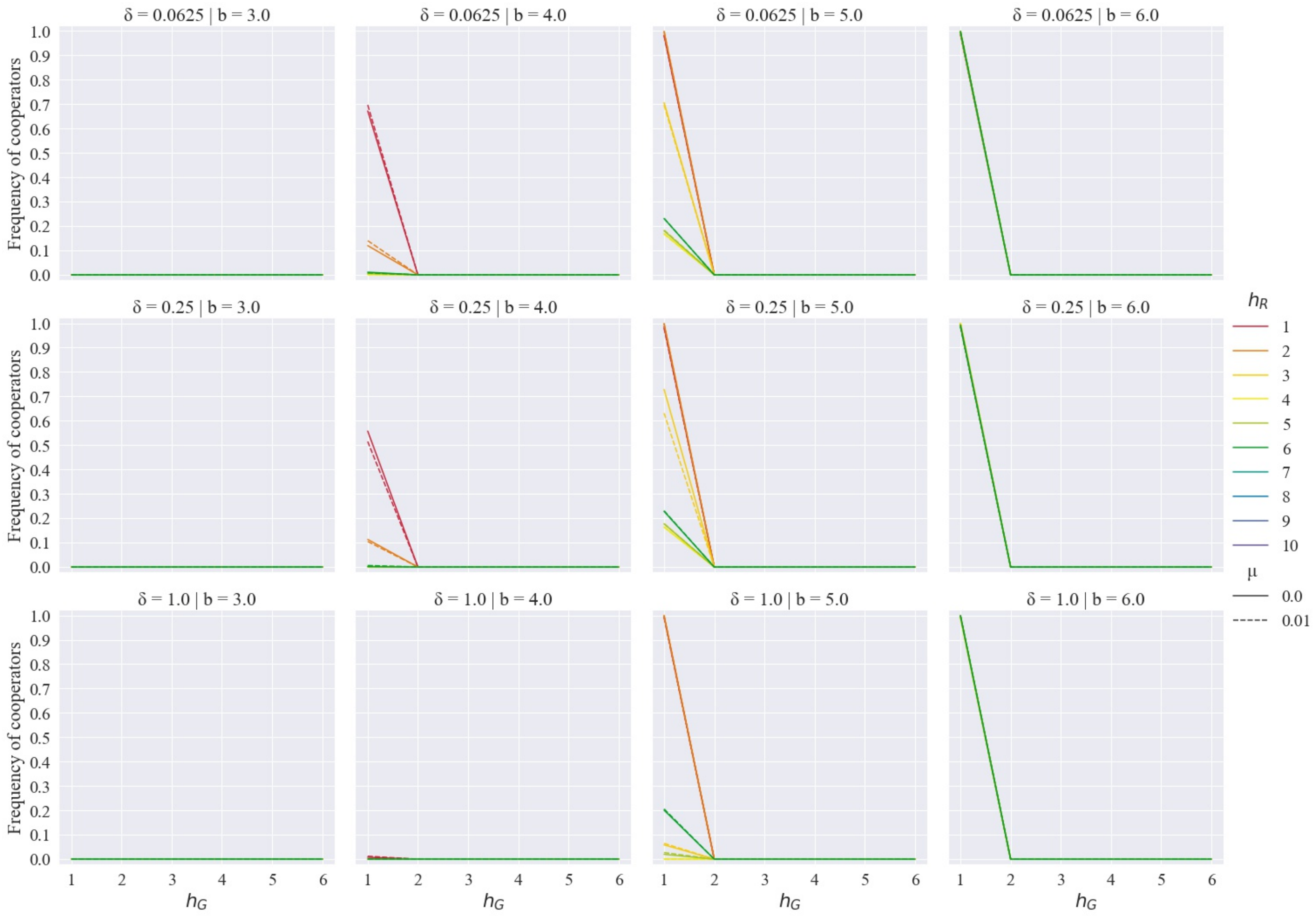

Figure 21: Random network, PGG, IM (X-axis: h_G)

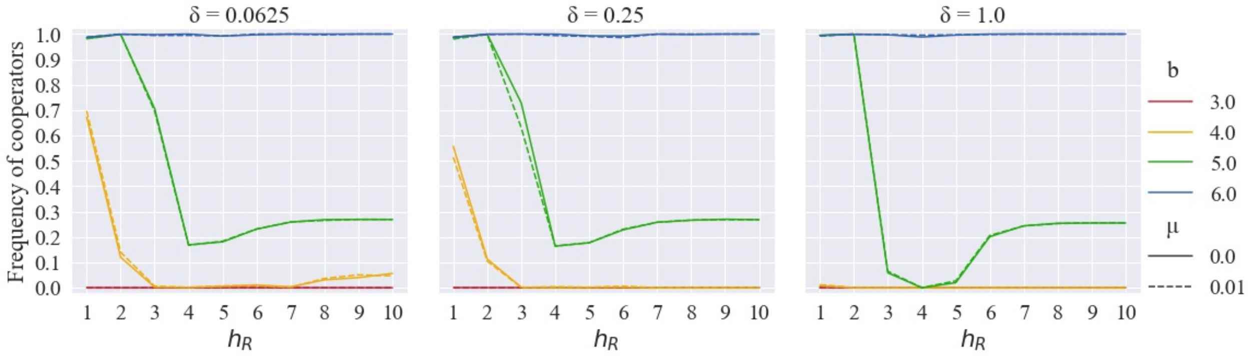

Figure 22: Random network, PGG, IM ($h_G = 1$, X-axis: h_R)

1.2.7 Regular network, PGG, BD

Cooperation rarely evolves under these conditions.

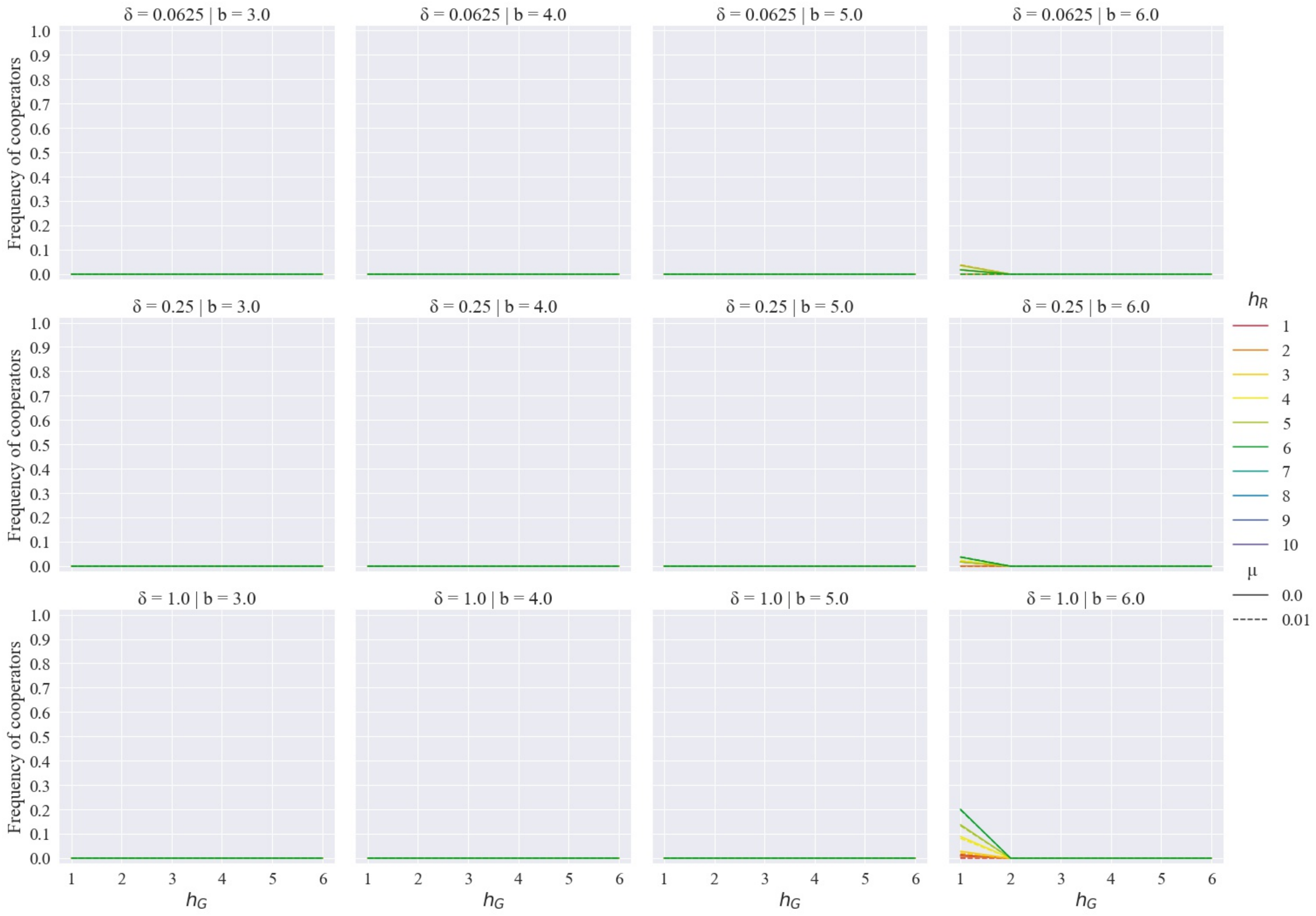

Figure 23: Regular, PGG, BD

1.2.8 Regular network, PGG, DB

Figure 24 and Figure 25 represent the same results but from different perspectives on the x-axis. Figure 24 illustrates that cooperation rarely evolves when the interaction scope is greater than 2. Figure 25 illustrates that cooperation rates are not stable in response to variations in b and h_R.

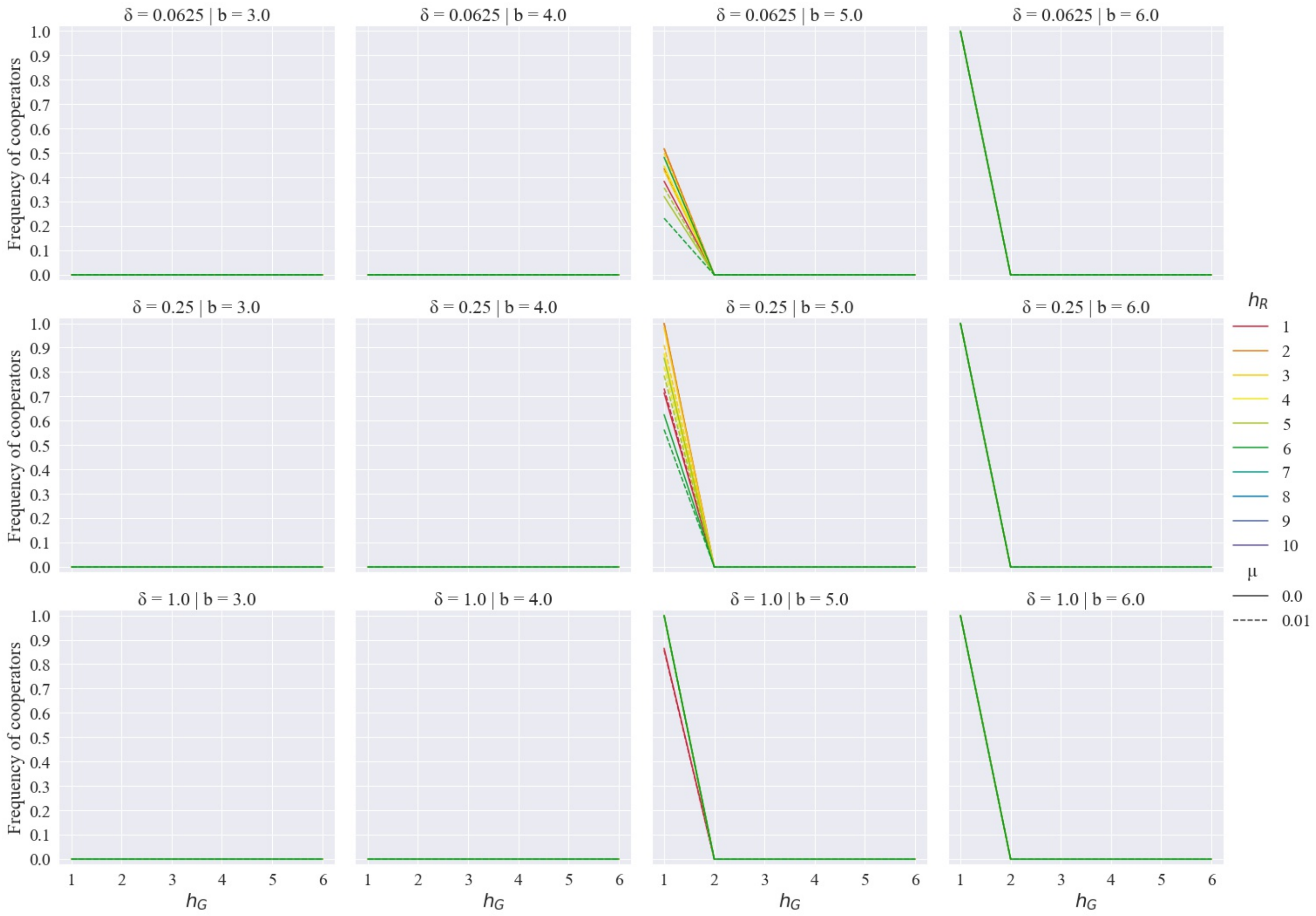

Figure 24: Regular, PGG, DB (X-axis: h_G)

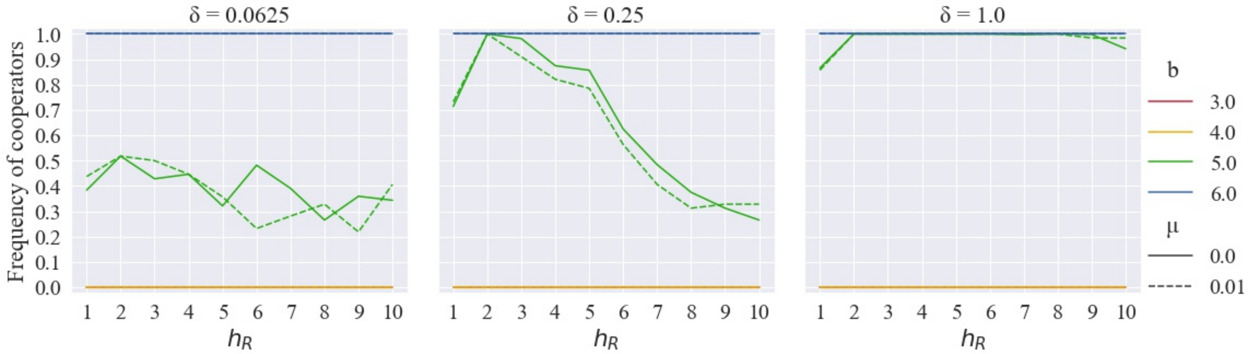

Figure 25: Regular, PGG, DB ($h_G = 1$, X-axis: h_R)

1.2.9 Regular network, PGG, IM

Figure 24 and Figure 25 represent the same results but from different perspectives on the x-axis. Figure 24 illustrates that cooperation rarely evolves when the interaction scope is greater than 2. Figure 25 illustrates that cooperation rates are not stable in response to variations in b and h_R.

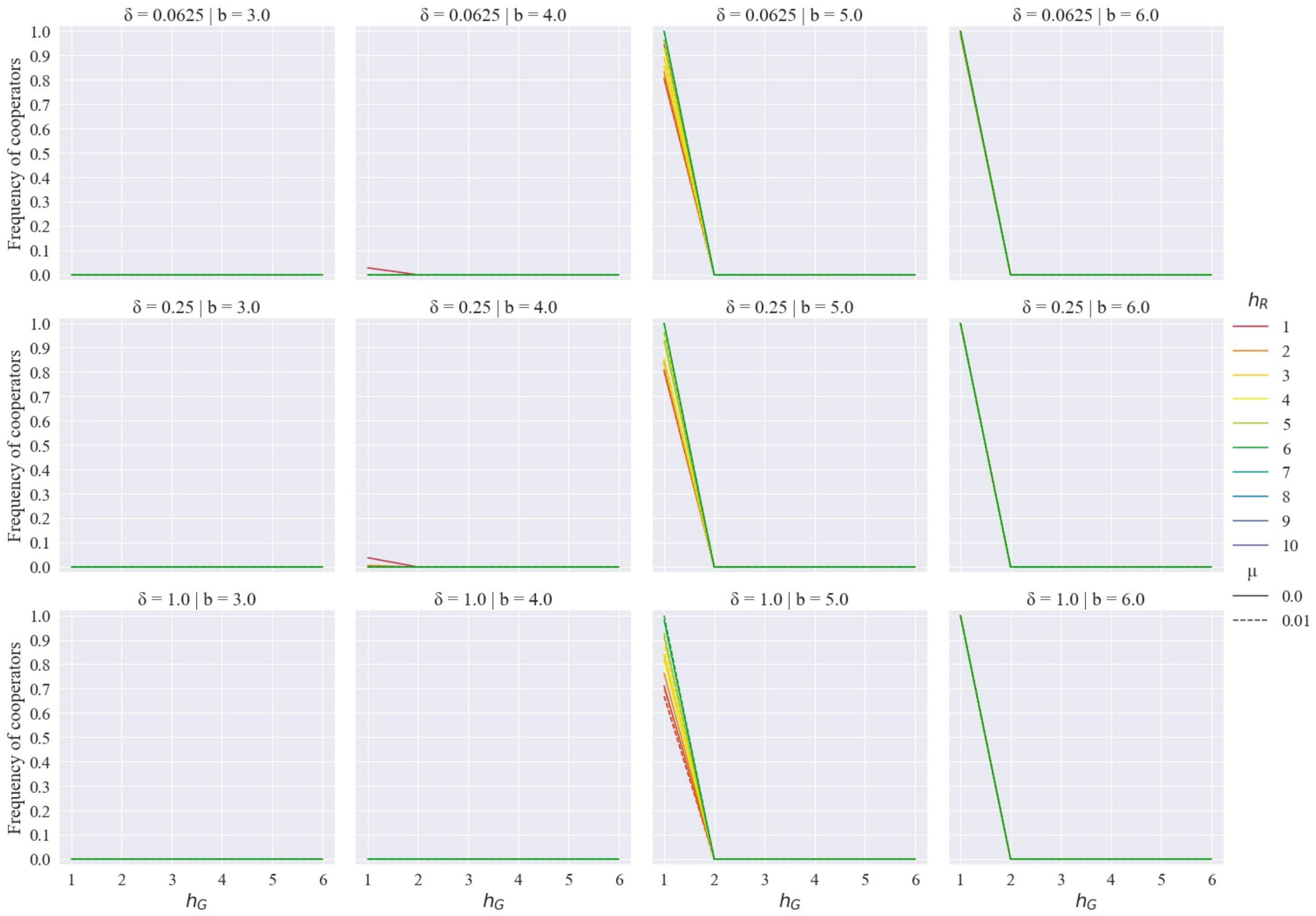

Figure 26: Regular, PGG, IM (X-axis: h_G)

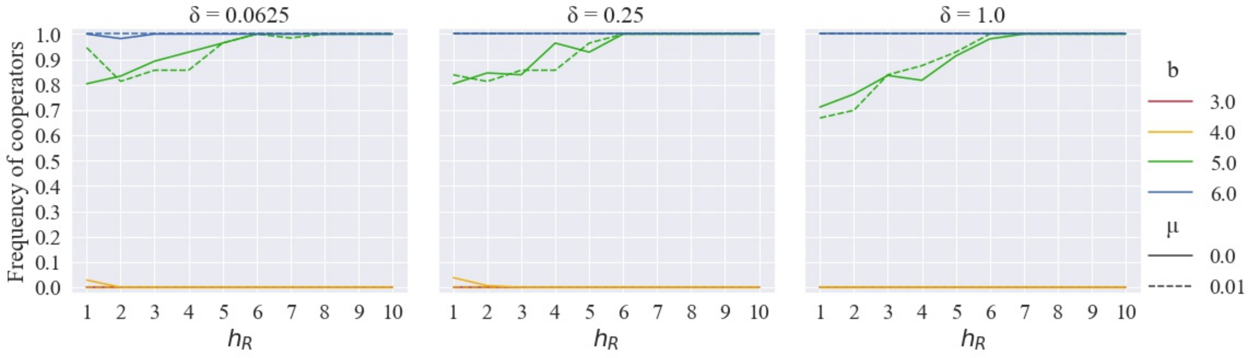

Figure 27: Regular, PGG, IM ($h_G = 1$, X-axis: h_R)

2 Expanded network features

The "Model" section in the main text explains how to expand the network, but what are the feature values of the resulting network? In the following section, we review the features of the expanded networks, including the degree distribution, average degree, average cluster coefficient, and average distance. Three types of network structures are used as a base: scale-free network (average degree $\bar{k} = 4$), random network ($\bar{k} = 4$), and regular network (degree $k = 4$). The number of nodes (N) is 1,000.

Each graph shows changes in the following features of the expanded networks with variations in expansion hop count: a. Degree distribution, b. Average degree, c. Average clustering coefficient, d. Average distance.

2.1 Features of expanded scale-free networks

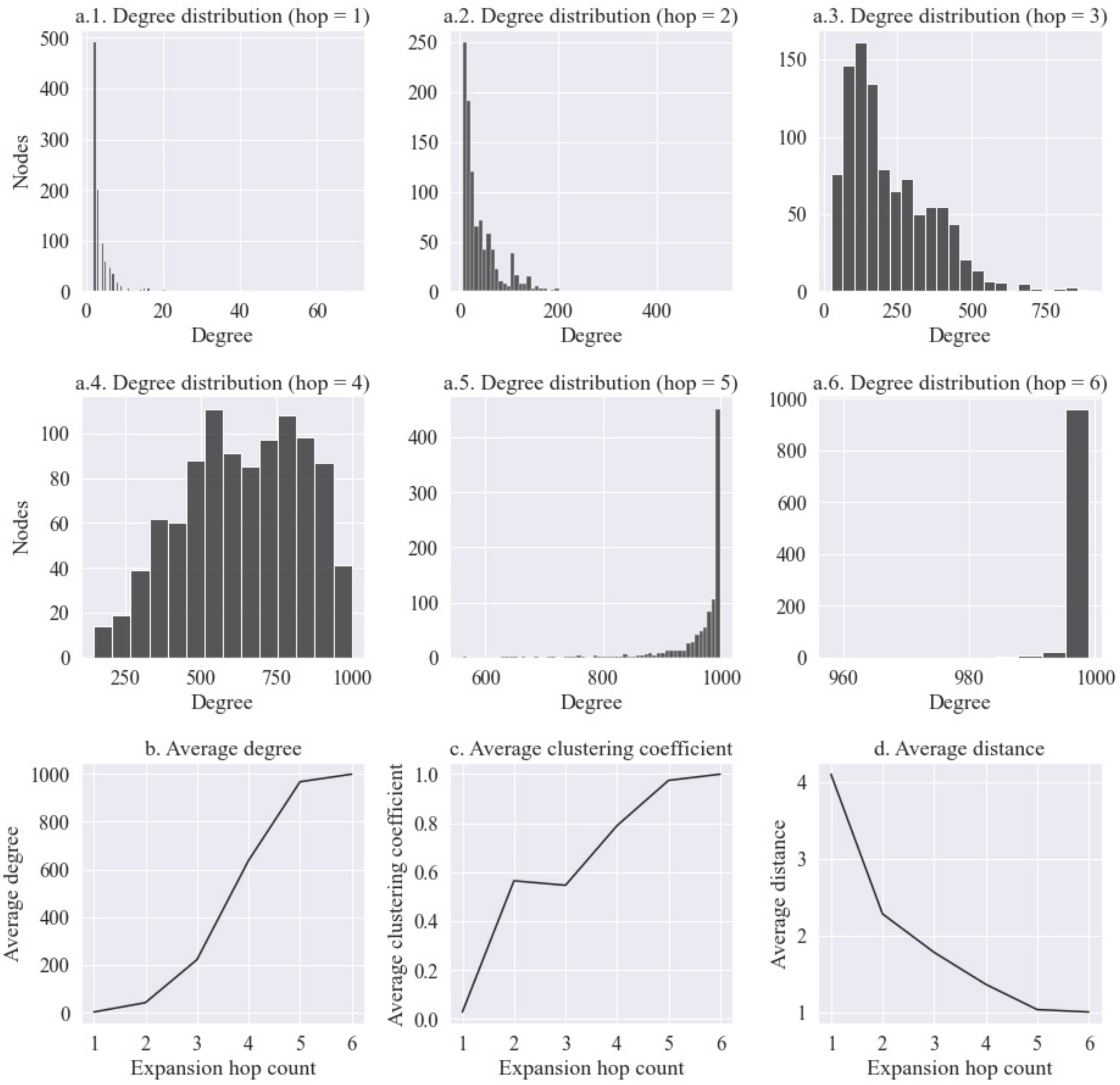

Figure 28: Features of expanded scale-free networks

It is well known that the degree distribution of a scale-free network follows the power law distribution, and in Figure 28a.1, it can be confirmed that many nodes have only a few edges, while a few nodes have a large number of edges. As the number of expansion hops increases, the power law distribution gradually collapses, and the network becomes an almost complete network (fully connected network) at hop = 6. Increasing the hop count beyond 6 does not change the network structure. The average clustering coefficient, shown in Figure 28c, exhibits a characteristic pattern: it increases sharply

when the hop count is increased from 1 to 2, decreases slightly when it is increased from 2 to 3, and then increases slowly thereafter.

2.2 Features of expanded random networks

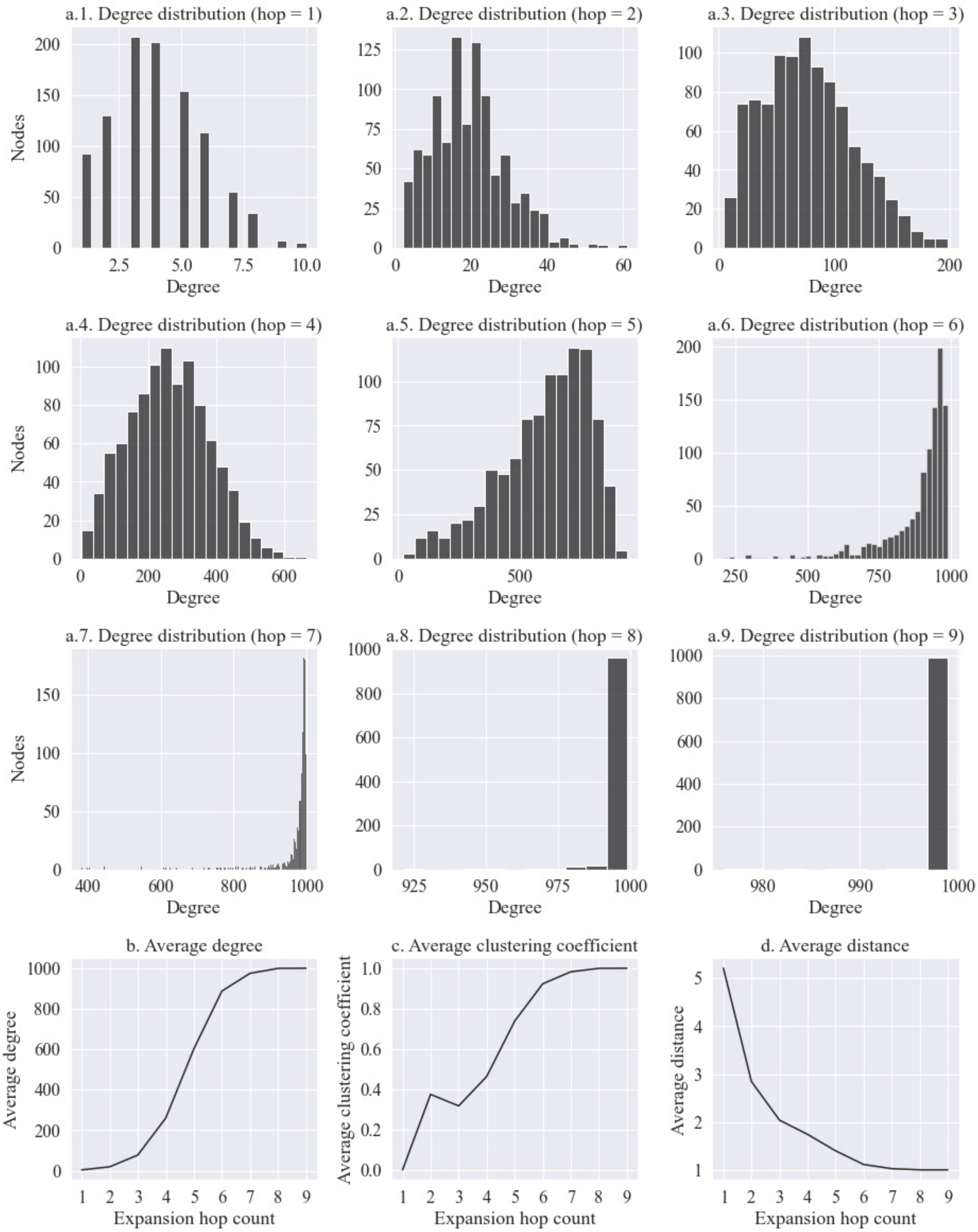

Figure 29: Features of expanded random networks

Figure 29a.1 shows that many nodes have a degree close to the average degree, and there are no nodes with an extremely high degree, unlike the scale-free network. This distribution follows the Poisson distribution. As the number of expansion hops increases, the degree distribution gradually collapses, and the network becomes an almost complete network at hop = 9. Increasing the hop count beyond 9 does not change the network structure. Similar to the scale-free network, the average clustering coefficient, shown in Figure 29c, exhibits a characteristic pattern: it increases sharply when the hop count is increased from 1 to 2, decreases slightly when it is increased from 2 to 3, and then increases slowly thereafter.

2.3 Features of expanded regular networks

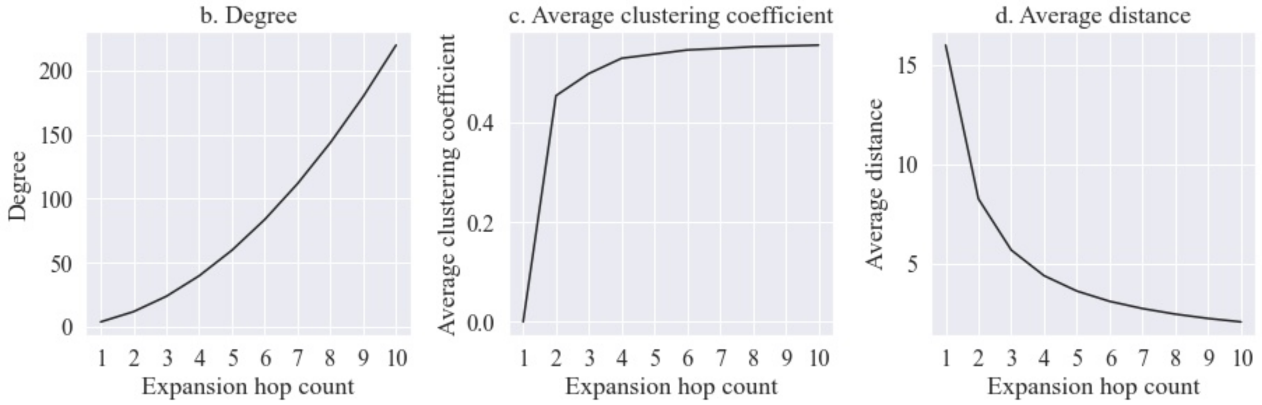

Figure 30: Features of expanded regular networks

The regular network maintains a regular network structure where all nodes have the same degree, even after expansion. Therefore, the degree distribution figures are omitted. The degree after expansion is represented by $k_h = 2h(h+1)$ ($\because k_{h+1} = k_h + 4h$). A regular network with 1,000 nodes becomes a complete network at $h = 22$. The average clustering coefficient (Figure 30c) increases rapidly when the hop count is increased from 1 to 2, similar to other network structures. However, unlike other network structures, there is no decrease when the hop count is increased from 2 to 3, and a gradual increase is observed after 2.
